# Genome-wide identification of Calcineurin B-Like (CBL) gene family of plants reveals novel conserved motifs and evolutionary aspects in calcium signaling events

**DOI:** 10.1186/s12870-015-0543-0

**Published:** 2015-08-06

**Authors:** Tapan Kumar Mohanta, Nibedita Mohanta, Yugal Kishore Mohanta, Pratap Parida, Hanhong Bae

**Affiliations:** School of Biotechnology, Yeungnam University Gyeongsan, Gyeongbook, 712-749 Republic of Korea; Department of Biotechnology, North Orissa University, Sri Ramchandra Vihar, Takatpur, Baripada, Mayurbhanj, Orissa 757003 India; Department of Botany, North Orissa University, Sri Ramchandra Vihar, Takatpur, Baripada, Mayurbhanj, Orissa 757003 India; Center for studies in Biotechnology, Dibrugarh University, Dibrugarh, 786004 Assam India

**Keywords:** CBL, CPK, Palmitoylation, Myristoylation, Evolution

## Abstract

**Background:**

Calcium ions, the most versatile secondary messenger found in plants, are involved in the regulation of diverse arrays of plant growth and development, as well as biotic and abiotic stress responses. The calcineurin B-like proteins are one of the most important genes that act as calcium sensors.

**Results:**

In this study, we identified calcineurin B-like gene family members from 38 different plant species and assigned a unique nomenclature to each of them. Sequence analysis showed that, the CBL proteins contain three calcium binding EF-hand domain that contains several conserved Asp and Glu amino acid residues. The third EF-hand of the CBL protein was found to posses the D/E-x-D calcium binding sensor motif. Phylogenetic analysis showed that, the CBL genes fall into six different groups. Additionally, except group B CBLs, all the CBL proteins were found to contain N-terminal palmitoylation and myristoylation sites. An evolutionary study showed that, CBL genes are evolved from a common ancestor and subsequently diverged during the course of evolution of land plants. Tajima’s neutrality test showed that, CBL genes are highly polymorphic and evolved via decreasing population size due to balanced selection. Differential expression analysis with cold and heat stress treatment led to differential modulation of OsCBL genes.

**Conclusions:**

The basic architecture of plant CBL genes is conserved throughout the plant kingdom. Evolutionary analysis showed that, these genes are evolved from a common ancestor of lower eukaryotic plant lineage and led to broadening of the calcium signaling events in higher eukaryotic organisms.

**Electronic supplementary material:**

The online version of this article (doi:10.1186/s12870-015-0543-0) contains supplementary material, which is available to authorized users.

## Background

**Fig. 1:**
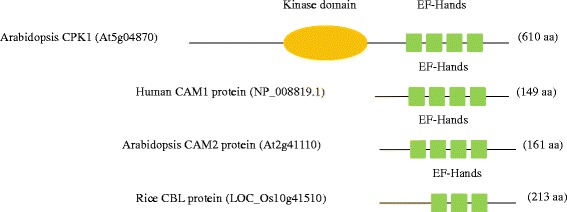
General structure of different calcium binding sensor protein. (**a**) *An Arabidopsis thaliana* CPK protein contain kinase domain and four calcium binding EF-hands, (**b**) human calmodulin (CaM) protein contains four EF hands, (**c**) *Arabidopsis thaliana* CaM2 protein contains four EF-hands, (**d**) rice CBL protein contains three EF-hands. From this figure it is clear that, CaM protein contain four calcium binding EF hands where as CBL protein contains three. The human CaM protein is shown here to identify the exact similarity between human and plant CaM protein and differences between plant CaM and CBL protein. The proteins were scanned in SCAN PROSITE (http://prosite.expasy.org/scanprosite/) software to check for the presence of calcium binding EF- hands

In various biological processes, calcium signals play a vital role as intracellular secondary messengers because of their strong homeostatic mechanism, which maintains an intracellular free Ca^2+^ concentration [1]. The concentration of calcium ions varies from 30 to 400 nM in resting cells and in millimolar range in organelles [2–4]. For cytosolic Ca^2+^ ion to be transported from cytosol to other parts of the cell, a low cellular level needs to be maintained. This can be achieved through the action of Ca^2+^-ATPase pump, which transports Ca^2+^ ions out of the cell across the plasma membrane, and sarco-endoplasmic reticulum Ca^2+^-ATPases that pump Ca^2+^ into the lumen of the endoplasmic reticulum [3]. It has been reported that, once cells began to use high-efficiency phosphate compounds as metabolic currency, they faced great challenges in maintaining low levels of intracellular Ca^2+^ [5] to prevent precipitation of calcium and phosphate salt in the cytosol, which ultimately forms a solid, bone-like structure. Since Ca^2+^ ion is a versatile signaling ion, it plays different roles across signaling cascades to regulate gene expression in plants [6]. Indeed, Ca^2+^ signals are important regulator of growth, development, and biotic and abiotic stresses in plants [7]. The signaling information encoded by Ca^2+^ ions is decoded and transmitted by calcium sensors of Ca^2+^-binding proteins [8, 9]. Such sensors binds Ca^2+^ ion and changes their conformation in a Ca^2+^ dependent manner in the presence of high levels of Mg^2+^ and monovalent cations [1, 10]. Some of the calcium sensor includes (i) calcium dependent protein kinases (CPKs), (ii) calmodulines (CaMs) and (iii) calcineurin B-like proteins (CBLs) [7, 11]. The CPKs are monomeric proteins with unique structures that contain five domains, the (i) N-terminal variable domain, (ii) kinase domain, (iii) an auto-inhibitory domain, (iv) a regulatory domain and (v) C-terminal domain. The regulatory domain of CPK is characterized by the presence of four Ca^2+^ binding EF (elongation factor)-hands. The EF-hands are calcium sensors characterized by the presence of a conserved Asp (D) or Glu (E) residue [7]. The EF-hand motifs are highly conserved, with a helix-loop-helix structure of 36 amino acid residues in each EF-hand. Unlike CPKs, CaMs and CBLs are small proteins that lack effector kinase domain (Fig. 1). The CaMs contain four Ca^2+^ binding EF-hands, whereas CBL contains three (Fig. 1) [12]. To transmit Ca^2+^ signals, CPKs, CBLs and CaMs interact with their target proteins, and regulate their gene expression [13]. These target proteins are may be protein kinases, metabolic enzymes, or cyto-skeletal associated proteins. The CIPKs (CBL-interacting protein kinase) are important target proteins of CBLs [14].

Although a great deal of effort has been made to investigate of the role of CBL genes, there has been very little effort made to determine the exact characteristics of these genes. Therefore, in this study, we identified CBL gene family members from 38 different plant species and assigned a unique nomenclature system to them. Additionally, we investigated the gene expression, genomics, phylogenetics and evolutionary aspects of these CBL genes.

## Results and discussion

### Nomenclature of CBL genes

To date, different members of specific gene families have been named according to the serial number by which they were identified. If no CBL gene has been identified for a given plant species to date, the first one identified is named CBL1, the next one as CBL2 and so on, regardless of the orthologous sequence similarity with the known counterpart genes. The volume of genomic sequence data are increasing daily, providing an excellent platform for genomics study. However, lack of a systemic nomenclature system for specific genes or gene families has led to confusion and difficulty in understanding the ever increasing genomic information. For example, the AtCBL1 gene differentially regulates salt, drought, and cold responses in *Arabidopsis* [15], but it is not clear whether the OsCBL1 gene also confers the same functionality. In principle, sequence similarity confers the structural similarity and structural similarity confers the functional similarity of a gene [16, 17]. Accordingly, AtCBL1 and OsCBL1 may confer more or less similar function. However, lack of a proper nomenclature system makes it very difficult to understand its function properly. Orthology lends the legitimacy to transfer functional information from an experimentally characterized protein to an uncharacterized one [18, 19]. Accordingly, an orthology based nomenclature system was adopted to name all CBL genes identified during this study as proposed by different researchers [7, 20–23]. In this system, *Arabidopsis thaliana* and *Oryza sativa* CBL protein sequences were taken as orthologous query genes. In the naming system, the first letter of the genus was kept upper case and the first letter of the species was kept lower case followed by CBL and then *A. thaliana* or *Oryza sativa* based CBL gene number. The monocot plants were named according to *O. sativa*, while dicot and other plants were named according to *A. thaliana*. In the case of monocot plants, the CBL gene number was assigned according to the orthologous gene of *Oryza sativa.* If more than one ortholog was found in a particular species, additional numbers followed by a hyphen were used to distinguish between paralogs. When the first letter of the genus and species of an organism coincided with another organism, the first letter of the genus was kept constant and the first, second, third or fourth letter or including the first, second, third and fourth letter of the species were taken into consideration. For example, the CBL gene of *Capsella rubella* was named as CrCBL, while *Chlamydomonas reinhardtii* was named as CreinCBL. In this case, both the letter of the genus and species name coincided with each other; therefore, the CBL gene of *C. reinhardtii* was denoted as CreinCBL. This nomenclature system can also provide information about the related orthologous species. The unique orthologous gene of one species may resemble the orthologous counterpart gene of another species and have undergone similar cellular function. The same approaches are usually used to predict the potential function for a newly sequenced gene and its protein product. It is very difficult to investigate the roles of all CBL genes in all plant species with different functional aspects. Therefore, the orthology based nomenclature system of the CBL gene will help to provide the basic information required for the counterpart orthologous gene.

### Genomics of CBL genes

The genome of a species is regarded as a bag of genes that contain all information’s necessary to bridge the gap between genotype and phenotype [24]. In the next decade, the genome sequences of virtually all angiosperms as well as important green algae, bryophytes, pteridophytes and gymnosperms will be completed. These genome sequences will become valuable tools that can provide a powerful framework for relating genome-level events to decipher the morphological and physiological variations that have contributed to colonization from aquatic habitats to land habitats. Genome-wide analysis of CBL genes across 38 different plant species revealed the presence of 328 CBL genes (Table 1). Among these, *G. raimondii* was found to contain the highest number of CBL genes (13) among higher land plants. The lower algae like *Chlamydomonas* and *Micromonas* contain only 2 and 3 CBL genes, respectively, in their genome. The bryophyte plant, *Physcomitrella patens*, and the pteridophyte plant, *Selagnella moellendorffii*, only encodes four CBL genes. The numbers of CBL genes found in *P. patens* is in accordance with the study of Kleist *et al.* [25]. The model gymnosperm plant, *Picea abies*, encodes 13 CBL genes. The genome size of an organism varies from species to species (Table 1). Among the monocot plant, *Zea mays* has the biggest genome (2500 Mbs) and encodes for 9 CBL genes where as among the dicot plants, *Glycine max* has the biggest genome (975 Mbs) and encodes for 9 CBL genes. The genome size of gymnosperm plant *Picea abies* is 1960 Mbs and encodes for 13 CBL genes. Similarly, the dicot plant *Capsella rubella* has the smallest genome (134.8 Mbs) and still contains 9 CBL genes in its genome. From this study, it is clear that, there is no correlation between the genome size and number of CBL genes in plants. In the case of blue green algae *Micromonas pusila*, its genome size is 22 Mbs and still contains 3 CBL genes whereas, the genome size of *Chlamydomonas reinhardtii* is 118.8 Mbs and only contains 2 CBL genes. The presence of specific numbers of CBL genes in its genome is independent of genome size and it might be correlated with functional evolutionary requirements of the plant. All CBL genes identified during this study contains only three calcium binding EF-hands. In our investigation, we did not find any CBL genes from green algae species *Coccomyxa subellipsoidea*, *Ostreococcus lucimarinus* or *Volvox carteri*. The CBL genes contain a maximum of six, seven, eight or nine introns in their gene; while only a few CBL genes are intronless (Additional file 1). The CBL genes of *Picea abies* are intronless. Other lower eukaryotic intronless CBL genes found during this study are from *M. pusila* (MpCBL2), *P. patens* (PpCBL3-3) and *S. moellendorffii* (SmCBL5), while higher eukaryotic intronless CBL genes were found from *S. lycopersicum* (SlCBL3-3) and *S. tuberosum* (StCBL3-3) (Additional file 1). The CBL gene of *F. vesca* FvCBL4 was found to be the largest CBL gene and posses an ORF (open reading frame) of 3048 nucleotides that encodes for 1015 amino acids. Similarly, the CBL gene of *M. domestica* MdCBL5 encodes the smallest CBL gene and that contains only 426 nucleotides ORF that encodes for 141 amino acids. The genome of *Z. mays* is the largest one, containing only nine CBL genes, whereas the genome of *M. pusila* is smallest one, with only two CBL genes. However, as shown in Table 1, larger genome size is not directly proportional to more CBL gene numbers. The molecular weights of CBL proteins are vary from 12.774 (PaCBL10) to 115.266 (FvCBL4) kDa, while the isoelectric point (pI) are ranges from 4.02 to 9.61. The majority of CBL proteins are acidic (Additional file 2). Based on the average amino acid composition of CBL proteins, the abundance of most important calcium sensing amino acids, Asp (D) and Glu (E) were found to be 8.07 and 8.94, respectively (Table 2). The average abundance of Trp and Cys amino acids in CBL proteins were 0.62 and 1.27, respectively.

**Table 1:** Genomic information of CBL genes in plants

Sl. no.	Name of plant species	Type of organism	Genome size (Mbs)	No. of CBL genes
1	*Aguilegia coerulea*	Dicot	302	5
2	*Arabidopsis thaliana*	Dicot	135	10
3	*Brachypodium distachyon*	Monocot	272	9
4	*Brassica rapa*	Dicot	283.8	14
5	*Capsella rubella*	Dicot	134.8	9
6	*Carica papaya*	Dicot	135	4
7	*Chlamydomonas reinhardtii*	Green algae	118.8	2
8	*Citrus clementina*	Dicot	301.4	7
9	*Citrus sinensis*	Dicot	319	8
10	*Cuccumis sativus*	Dicot	203	7
11	*Eucalyptus grandis*	Dicot	691	12
12	*Fragaria vesca*	Dicot	240	6
13	*Glycine max*	Dicot	975	9
14	*Gossipium raimondii*	Dicot	761.4	13
15	*Linum usitatissimum*	Dicot	318.3	12
16	*Malus domestica*	Dicot	881.3	11
17	*Manihot esculenta*	Dicot	760	9
18	*Medicago truncatula*	Dicot	257.6	11
19	*Micromonas pusilla*	Green algae	22	3
20	*Mimulus guttatus*	Dicot	321.7	9
21	*Oryza sativa*	Monocot	372	11
22	*Panicum halii*	Monocot	453	8
23	*Panicum virgatum*	Monocot	1358	10
24	*Phaseolus vulgaris*	Dicot	521.1	10
25	*Physcomitrella patens*	Moss	480	4
26	*Picea abies*	Gymnosperm	1960	13
27	*Populous trichocarpa*	Dicot	422.9	11
28	*Prunus persica*	Dicot	451.9	7
29	*Ricinus communis*	Dicot	400	8
30	*Selaginella moellendorffii*	Pteridophyte	212.6	4
31	*Setaria italica*	Monocot	405.7	7
32	*Solanum lycopersicum*	Dicot	900	11
33	*Solanum tuberosum*	Dicot	800	12
34	*Sorghum bicolor*	Monocot	697.5	8
35	*Thellugenella halophila*	Dicot	238.5	9
36	*Theobroma cacao*	Dicot	330.8	7
37	*Vitis venifera*	Dicot	487	9
38	*Zea mays*	Monocot	2500	9

The splice variants of CBL genes were not included in this study. From the table, it indicates that number of CBL genes of a species is not directly proportional to its genome size

**Table 2:** Average amino acid composition of CBL proteins in plants

Amino acids	Average amino acid composition of CBL gene	Energy cost for amino acid synthesis
Ala	5.99	11.7
Cys	1.27	24.7
Asp	8.07	12.7
Glu	8.94	15.3
Phe	7.97	52.0
Gly	3.96	11.7
His	2.12	38.3
Ile	6.25	32.3
Lys	7.03	30.3
Leu	10.84	27.3
Met	2.24	34.3
Asn	3.79	14.7
Pro	3.29	20.3
Gln	2.89	16.3
Arg	4.54	27.3
Ser	7.18	11.7
Thr	4.75	18.7
Val	6.19	23.3
Trp	0.62	74.3
Tyr	1.98	50.0

From the table we can see that, more the energy required for synthesizing a specific amino acid, the abundance of that amino acid is very less in the CBL protein

The genome sizes of plants are remarkably diverse and vary from species to species, with sizes that range from 63 (*Genlisea aurea*) to 149,000 Mbs (*Paris japonica*), divided into *n* = 2 to approximately *n* = 600 chromosomes and remains constant within a species [26]. In this study, we found that the dicot plant *Arabidopsis thaliana* and *Carica papaya* (135 Mbs) have the smallest genome size, whereas in the monocot plant *Zea mays* (2500 Mbs) have the largest genome size among the higher plants. The lower eukaryotic algae, *Micromonas pusila* (22 Mbs), contains the smallest genome among the investigated species. The gymnosperms are characterized by the presence of a very large genome (up to 35,000 Mb), and *Picea abies* contains 1960 Mbs genome [27]. Despite their larger genome, gymnosperms do not have higher numbers of chromosomes, with the number ranging between 2n = 2x = 14-28. *Arabidopsis* genome sequencing was initiated based on the thinking that genes and gene sequences of *Arabidopsis* would be similar to those of other plants, which was later found to be true; however, the number of protein coding genes varied significantly. This also found to be true in this study as the numbers of protein coding genes vary in a specific gene family of a specific plant. The nuclear DNA of plant consists of a low copy number of coding sequences, introns, promoters and regulatory DNA sequences [26]. In this study, the majority of CBL genes were found to have either six, seven or eight introns within it, suggesting, the presence of intron number within a specific gene family varies from species to species, as well as in their counterpart orthologous gene(s).

It is well known that individual genes and entire genome can vary significantly in nucleotide compositions [28, 29]. The mutational process and relationship between the primary structure and function of a protein is considered as the major determinants of amino acid composition and rate of protein evolution [30]. The natural selection events usually enhances the protein specificity and stability by favouring codons that encodes particular amino acids in a specific genic region [31]. However, metabolic constraints on protein structure and composition could include the energetic cost of amino acid biosynthesis. The biosynthesis of aromatic amino acids like Trp requires higher energy (74.3 unit) and hence the average abundance of Trp amino acid per CBL gene is only 0.62 amino acids [30]. High energy is required to synthesize Trp amino acids, so plants have encoded only 0.6 amino acids per CBL protein to avoid extra energy expense. Similarly, 12.7 and 15.3 units of energy is require for biosynthesis of Asp and Glu amino acid, respectively. Biosynthesis of Asp and Glu amino acid is relatively less costly; hence, plants encoded 8.07 and 8.94 amino acids, respectively, per CBL protein. As plants use a substantial amount of energy for biosynthesis of amino acids, there is an advantage to encode less costly amino acid in their protein [30].

### Conserved EF-hands

**Fig. 2:**
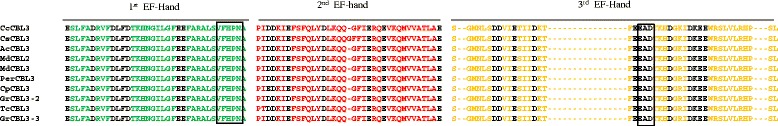
Figure showing the presence of three EF-hands in CBL protein. The green color indicates the 1st EF-hand, red color indicates 2nd EF-hand and orange color indicates 3rd EF-hand. The presence of conserved Asp (D) and Glu (E) amino acids in EF-hands of CBL protein confers binding of calcium ions. Among the three EF-hands, 3rd EF-hand of CBL protein contains E-E-x-D and D-x-D/E calcium binding motifs. All the conserved amino acids (D and E) and motifs present in EF-hands were marked in black color. In the first EF-hand Glu (E) amino acid is conserved at 1, 23 and 24 position and Asp (D) amino acid is conserved at 6, 10, and 13 positions. In second EF-hand Asp/Glu amino acid is conserved at 3, 4 and 7 position but Asp amino acid is significantly conserved at14 position. The Glu amino acid is most significantly conserved at 22 and less significantly conserved at 25 positions. At position 36, Glu amino acid is significantly conserved. In 3rd EF-hand D-D-x-x-E motif is present at 7, 8, 9 10 and 11 position. Asp (D) amino acid is conserved at 15 and 26 position. The E-E-x-D motif is present at 19, 20, and 21 and 22 and D-x-E-E motif is present at 30, 31, 32 and 33 position respectively. The abundance of Asp and Glu amino acids are much more in 3 EF-hand when compared to 1 and 2 EF-hand

Multiple sequence alignment of the CBL proteins revealed the presence of several new conserved domains and motifs. The CBL proteins of the plant kingdom contain only three EF-hand domains and are conserved. Overall, each EF-hand is 36 amino acids in length and has a helix-loop-helix structure [32]. Each helix loop contains 12 amino acids within it; hence, each EF-hand contains 36 amino acids. Multiple sequence alignment revealed that, Asp (D) amino acid is less significantly conserved at position 7 and 11 in the first EF-hand, but most significantly conserved at position 14 (Fig. 2, Additional file 3). Additionally, Asp (D)/Glu (E) amino acids are conserved at positions 22 and 25. Several other amino acids are also conserved in the first EF-hands. However, the major focus was given to calcium sensing Asp (D) and Glu (E) amino acid. If we consider the presence of conserved domains in CBL proteins, there is a conserved V-F-H-P-N domain at the end of the first EF-hand (Fig. 2). In the second EF-hand, Asp/Glu amino acids are slightly conserved at the 3, 4 and 7 position, but Asp is significantly conserved at the 14 position (Fig. 2). The Glu amino acid is most significantly conserved at position 22 and is less significantly conserved at position 25. The Glu amino acid is also significantly conserved at position 36. In the third EF-hand, Asp amino acid is conserved at position 7, 8 and 14; while Glu is conserved at position 11, 19, 20, 21 and 22 (Fig. 2). The Asp and Glu amino acids are present as a D/E-x-D motif at position 20, 21 and 22 of the third EF-hand. Another motif, D-x-E-E, is present at position 30, 31, 32 and 33 in the third EF-hand. Taken together, these findings indicate that, the third EF-hand contains the maximum Asp and Glu amino acids within it. In EF-hand loop, the calcium ion is coordinated in a pentagonal bi-pyramidal configuration. Earlier study in CPK EF-hand revealed that, six amino acid residues are involved in binding of calcium ion in each EF-hands and are present at position 1, 3, 5, 7, 9 and 12 [7]. These residues are denoted by X, Y, Z, −Y, −X and –Z. The invariant Glu or Asp amino acid at position 12 provides two molecules of oxygen for liganding Ca^2+^ (bidentate ligand) ion [7]. The position 1 (X), 3 (Y) and 12 (−Z) are the most conserved and plays critical role in calcium binding. In case of CBLs, the presence of Asp or Glu amino acids at position 7, 14 and 22 are very critical for binding calcium ion while other conserved Asp and Glu amino acids might provides the accessory affinity sites for strong calcium binding.

**Fig. 3:**
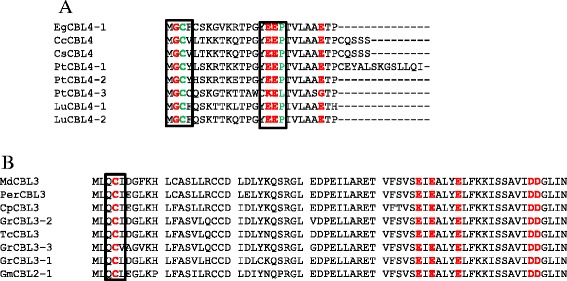
The N- and C-terminal conserved amino acids of CBL proteins. (**a**) indicates conserved E-E/D-P amino acid motif (box) in N-terminal region of CBL genes. This motifs is present upstream to calcium binding EF-hands. (**a**) indicates group D CBL gene specific and present at 16, 17 and 18 position from the start site. Conserved sequences of D/E-x-E/D in (**b**) represent group A CBL gene specific and present at 31, 32 and 33 position from the start site (in the (**b**), position of amino acid indicated in box should be read as 31, 32 and 33 position from start site)

**Fig. 4:**
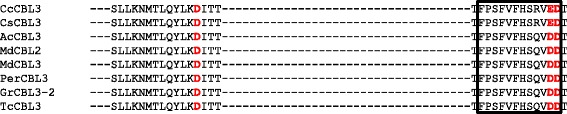
Presence of conserved P-S-F-V-F-x-S-E-V-D-E domain in C-terminal region of CBL proteins. To get more detail about conserved sequences, please see Additional file 3

There is a presence of an upstream region immediately adjacent to the first EF-hand of the CBL protein (Fig. 3). This up-stream region is not significantly conserved, but contain several calcium binding Asp and Glu amino acids (Additional file 3). The Group D CBL protein was found to contain conserved Asp and Glu at position 16, 17 and 18 (E-E/D-P) in the N-terminal region (Fig. 3a). In the group A CBL protein, there is a D/E-x-E/D motif present at up-stream of the first EF-hand (N-terminal region) (Fig. 3b). A less conserved domain E/D-D-P-E-X_4_-E-X_6_-E is present at the N-terminal region of the CBL protein (Additional file 3). In the C-terminal region, there is a conserved P-S-F-V-F-x-S-E-V-D-E domain present downstream of the third EF-hand (Fig. 4).

**Fig. 5:**
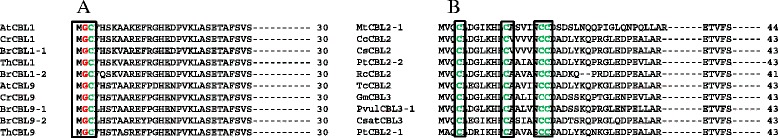
Conserved myristoylation and palmitoylation site of CBL Proteins. **a** indicates the presence of Gly (G) amino acid at second position (marked in red inside the box). The Gly amino acid at second position of CBL protein represents probable myristoylation site. **b** represents the presence of conserved Cys (C) amino acid residue at third position (marked in green inside the box). **a** also contains Cys amino acid at fourth position (marked in green inside the box). The presence of Cys amino acid at third, fourth, fifth or six position of CBL protein represent probable palmitoylation site. The Cys amino acid up to 25 position from start site is responssible for protein palmitoylation. The Lys amino acid is also a probable protein palmitoylation site, but in majority of cases it is found in prokaryotes

The organisms are able to recognize sense and respond to their environment to survive. In plants, sensing mechanisms are evolved in response to hormonal and environmental signals [33]. To elicit a cellular response, the perceived signal must be conveyed to its cellular machinery. One of the most important secondary messengers, Ca^2+^, perceives the stimulus and transduces it to the downstream protein to initiate Ca^2+^ mediated responses. The Ca^2+^ mediated stimuli causes plant to respond to hormone and external stimuli, which mediate and regulate diverse fundamental cellular processes such as cell division, cell elongation, cell differentiation, cell polarity, photo morphogenesis, plant defense and stress responses [31]. The CBL protein is one of the several calcium sensing protein families, including calcium dependent protein kinase (CPK) and calmodulins. The CPK protein contains a kinase domain as well as a regulatory domain that has four calcium sensing EF-hands. The acidic amino acids Asp (D) and Glu (E) present in the EF-hands are important calcium sensors [34]. The CBL proteins lack the kinase domain and contain only three calcium binding EF-hands. The CBL proteins of *Arabidopsis thaliana* and *Oryza sativa* were previously reported to contain four calcium binding EF-hands [35–37]. However, the scan prosite software study revealed that, CBL proteins of all plants contain only three calcium binding EF-hand domains (Figs. 1 and 5) [38]. Investigations of the CBL proteins of Kudla *et al.* [35], Batistic and Kudla [39] and Gu *et al.* [37] using the scan prosite software revealed that, all CBL proteins reported to have four EF-hands actually contained only three EF-hands. They reported that, in some cases CBL protein contains four EF-hands while in other they contain incomplete four EF-hands. The prosite analysis of data provided by Weinl and Kudla [40] shows that, *O. tauri* protein contains clear four EF-hands where as *S. moellendorfii* protein shows only three EF-hands. One CBL protein contains four EF-hands whereas other contains three EF-hands. This is very contradicting. This proves that, the data provided by Weinl and Kudla are contradictory. Some other data provided in this manuscript belongs to genus *Physcomitrella patens* (FJ901251, FJ901252, FJ901253 and FJ901254). Here the *P. patens* FJ901254 protein contains four EF-hands while other contains only three EF-hands. The CBL genes are present from single celled *Chlamydomonas* to the modern land plants. The *Chlamydomonas* is considered as the basal evolutionary lineage of photosynthetic green plant that evolved since 3500 million years ago, which is far earlier than the evolution of land plants. So, it is highly unlikely that genome(s) will encode for incomplete functional EF-hands for more than 3500 million years. Genomes are very specific in nature. They would either encode for complete EF-hand or would remove the incomplete one. But nothing has happened; because there is not presence of such incomplete EF-hands in CBLs. Evolutionary pressure cannot allow transfer of incomplete and non-functional EF-hand for millions of years. This proves that CBLs protein contain only three calcium-binding EF-hands, not four or incomplete four.

Although there have been significant advances in our understanding of CBL proteins, no studies are available regarding their conserved domains and motifs. In this study, we found that the calcium binding EF-hands are highly conserved and contains the E/D-x-D motif in the third EF-hand (Fig. 2). In addition to this motif, CBLs also contain several C-terminal downstream conserved motifs, specifically conserved Asp and Glu amino acids (Fig. 3a and b). The high proportion of Asp and Glu amino acids in CBLs provides an opportunity for the accommodation of Ca^2+^ ions.

### Myristoylation and palmitoylation sites

Protein myristoylation and palmitoylation are two important events necessary for protein trafficking, stability and aggregation [41]. Addition of myristic acid to N-terminal Gly amino acid leads to protein myristoylation, while addition of palmitic acid to N-terminal Cys amino acid leads to protein palmitoylation [42]. In most of the studied CBLs, N-terminal Gly amino acid is required for protein myristoylation and is conserved at the second position (Fig. 3). The N-terminal Gly amino acid in some other CBL proteins has been found to be conserved at the seventh position. Similarly, N-terminal Cys amino acid is required for protein palmitoylation and is conserved at the third position in group D CBL proteins (Fig. 3a) and at the fourth position in group A CBL proteins (Fig. 3b). The majority of group B CBLs don’t contain N-terminal Cys amino acids.

The protein palmitoylation is a widespread modification found in membrane bound protein that includes transmembrane-spanning protein synthesized in soluble ribosome [43]. In general, protein palmitoylation increases the affinity of protein for membrane attachment and therefore affects protein localization and function. Proteins that undergo palmitoylation include RasGTPase [44], Rho GTPase [45] and CDPKs [7]. The RasGTPase, Rho GTPase, and CDPKs contain N-terminal Cys residues at either the third, fourth or fifth position [46]. All the 24 *Arabidopsis* CPKs are predicted to have a myristoylation consensus sequence and contain at least one Cys residue either at fourth, fifth or sixth position [47]. This study revealed the presence of an N-terminal Cys residue at the third, fourth, fifth or sixth position in several CBLs (Figs. 3 and 5a and b). Except for group B CBLs (CBL10), all other group of CBL proteins (group A, C and D) contain the N-terminal Cys residue. These finding clearly demonstrates that, group B CBL protein does not undergo protein palmitoylation, and only selective CBL protein posse’s protein palmitoylation activity.

Co-translational addition of myristate to N-terminal glycine amino acid through amide bonds is known as myristoylation [42]. Except in group B CBLs, all other CBLs contain N-terminal glycine residues at the second position (Fig. 5a). Additionally, all CBLs (except group B CBLs) that contain N-terminal cysteine amino acid concurrently possess N-terminal Gly amino acid at the second position (Figs. 3 and 5). The N-terminal myristoylation promotes protein-membrane attachment and protein-protein interactions. Mutation in the N-terminal Gly-abolishes lipid modification and thus prevents membrane association [48]. Twenty-four of the *Arabidopsis* calcium sensing CDPK proteins were predicted to have the N-terminal myristoylation motif for membrane association. Among them, AtCPK2 has been experimentally confirmed to be myristoylated at the N-terminal Gly residue, and the first ten amino acids of the CPK protein are critical for localization to the ER (endoplasmic reticulum) membrane [49]. In majority of cases, N-terminal myristoylation and palmitoylation events are complement to each other. Both N-terminal myristoylation in the Gly amino acid at position 2 and palmitoylation in the Cys amino acid at position 4 and 5 have been validated experimentally in membrane bound OsCPK2 [48]. When N-terminal myristoylation was abolished by mutation at the Gly amino acid, the protein could no longer be palmitoylated, indicating that N-terminal myristoylation is the prerequisite for palmitoylation. Only protein myristoylation provides a weak affinity for membrane attachment, whereas palmitoylation and myristoylation provide very high affinity interactions [48].

### Phylogeny and evolution

**Fig. 6:**
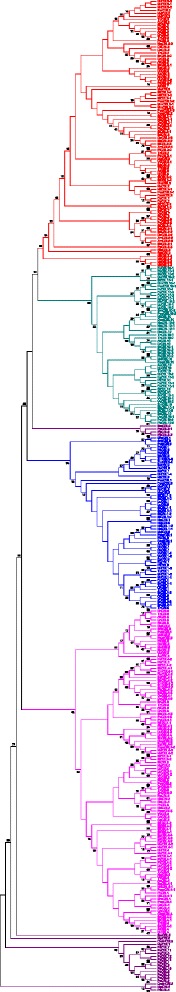
The phylogenetic tree of CBL proteins. The phylogenetic analysis shows that, CBL proteins are grouped into five different clades. The grouping of CBLs are done according to their presence from top to bottom in the phylogenetic tree and denoted in color mark; group A (red), group B (green), group C (blue), group D (fuschia) and group E and F (purple). Different CBL proteins distributed in different groups are; group A (CBL2, CBL3, CBL6, CBL7), group B (CBL10), group C (CBL1, CBL9), group D (CBL4, CBL5, CBL8), group E and F are lower eukaryotic specific CBLs. The phylogenetic tree was constructed using MEGA5 software. Statistical parameters used to construct the phylogenetic tree were as follows: test of phylogeny, bootstrap method; number of boot strap replicate, 2000; model/method, Jones-Taylor Thornton (JTT); missing data treatment, partial deletion; ML heuristic method, nearest neighbor-interchange (NNI) and branch swap filter, very strong. Detailed data of CBLs can be found in Treebase (Additional file 5), a database for phylogenetic knowledge (http://purl.org/phylo/treebase/phylows/study/TB2:S17414?x-access-code=1b88565e08ce238f8fc7928d2fa11a12&format=html)

Protein families are defined as groups of protein with more than 50 % pairwise amino acid sequence similarity [50]. Molecular evolution is generally studied at the level of individual gene or families of genes [51]. However, there are still no models that can infer gene family evolution to enable the estimation of the ancestral state. Phylogenetic analysis can be a powerful tool to infer the relationships among genes and analyze their evolutionary events [52]. Phylogenetic analyses of all CBL genes together revealed that they fall into six different groups (Fig. 6). Some lower eukaryotic specific CBL genes such as SmCBL9, PpCBL3-1, PpCBL9 and PpCBL3-2 are present as a cluster (group F) at the center of the phylogenetic tree, while group E CBLs are present at the distal end of the phylogenetic tree. The cluster of other CBL genes of higher eukaryotic plants (group A, B, C and D) was directly linked with the cluster of group F CBL genes (Fig. 6, Additional file 5). These findings indicate that CBL gene families of higher eukaryotic plants are derived from common ancestors of lower eukaryotic plants (Fig. 6). The lower eukaryotic plants are very simple, with unicellular to multi-cellular architecture. As complexity of an organism increases, it need to adapt from simpler aquatic habitats to complex terrestrial habitats, and hence the number of CBL genes per genome got increased [53]. This indicates that these CBL genes might have been evolved for some unique and specific function responsible for adaptation to complex lifestyles. The CBL genes of lower eukaryotic plants such as algae, *Physcomitrella*, *Selaginella* and *Pinus* are fall in group E and F. These genes are probably evolved independently during evolution. Some of the CBL genes (SmCBL9, PpCBL3-1, PpCBL9 and PpCBL3-2) of lower eukaryotic plants fall in the middle of the phylogenetic tree, while CBL genes of higher angiosperm plants are phylogenetically linked with the cluster of CBL genes of lower eukaryotic plants. These findings indicates that, CBL genes of modern plants may have derived from a common ancestor of lower eukaryotic plant [54]. The phylogenetic analysis revealed that, CBL2, CBL3, CBL6 and CBL7 fall in group A, CBL10 falls in group B, CBL1 and CBL9 fall in group C, and CBL4, CBL5 and CBL8 fall in group D. The lower eukaryotic CBL genes from *Selaginella* (SmCBL5), *Micromonas* (MpCBL2, MpCBL6), *Chlamydomonas* (CreinCBL8, CreinCBL9), and CBL genes of *Picea abies* fall in group E and F.

The significant similarities between the CBL gene sequences indicate that they arose relatively recently via gene duplication and might have similar or overlapping functions. The paralogous genes evolved due to the development of new function and provided the most probable role for adaptation. Gene duplication and diversification are considered to be the most important events in evolutionary biology. If a gene is duplicated from its original gene, the selective constraints become much lower for the extra copy, and it can evolve to have a slightly different function while the original function of the gene is kept in the other copy. Hence, gene duplication with subsequent diversification is one of the simplest ways to acquire new function. Because the role of the CBL gene is important for calcium sensing and there are several other calcium sensing gene families (CPK, CaM, *etc.*) present in the plant kingdom, duplicated genes are still being found for CBL genes. This may be due to the ploidy level, as well as some other aspects in different genomes. Some plant genomes that have undergone duplication during evolution contain few duplicated CBL genes including *Brassica rapa, Eucalyptus grandis, Glycine max, Gossipium raimondii* and *Medicago truncatula*.

### Tajima’s statistics

Tajima’s molecular test hypothesis explains the significance and rate of evolution [55]. Random analysis of CBL sequences was carried out in Tajima’s relative rate test and the *p-*value and *X*^*2*^*-*test was found to be significant (Table 3). Three random replicate analyses were carried out. In each analysis, three sequences were considered for the study by making them as group A, B and C. The first analysis contained sequences of MdCBL3 (group A), CsCBL3 (group B), and PerCBL3 (group C); the second analysis contained MeCBL3 (group A), BrCBL2-2 (group B), and PtCBL3 (group C); and the third analysis contained FvCBL10-1 (group A), BrCBL2-2 (group B), and MgCBL5 (group C). In the statistical analysis, the *p-*value was found to be 0.00666, 0.00284 and 0.00555 for the first, second and third analysis, respectively (Table 3). Similarly, the chi-square values for the first, second and third analysis was found to be 7.36, 8.91 and 7.69, respectively, with one degree of freedom (Table 3). These findings suggest that, the results presented herein are statistically significant. In Tajima’s test for neutrality, Tajima’s D value for CBLs was found to be 4.413697 (Table 4). In Tajima’s D-test, when D = 0, the average heterozygosity of a population becomes equal to the number of segregating sites. This occurred because the expected variation is similar to the observed variations [55, 56]. Hence, evolution of the population can be due to mutation-drift equilibrium, and there is no evidence of selection. When D < 0, the average heterozygosity is lower than the number of segregating sites [55, 56]. This indicates that, rare alleles are present at very low frequency and recent selective sweeps led to the expansion of the population size after recent bottleneck. When D > 0, the average heterozygosity is more than the segregating sites and can be considered as the presence of multiple alleles at high frequencies [55, 56]. This leads to balanced selection due to the sudden contraction in population size. Tajima’s negative D value signifies a very low frequency of polymorphism relative to expectation, indicating expansion in population by size via selective sweep or purifying selection. Tajima’s positive D value signifies a high frequency of polymorphism, indicating a decrease in population size by balancing selection. A Tajima’s D value greater than 2 or less than −2 is considered significant [55, 56]. In this study, Tajima’s D value is 4.413697 (Table 4), signifying that CBL genes have undergone high frequencies of polymorphism via decreasing population size due to balanced selection. Accordingly, the heterozygosity of CBLs is greater than the number of segregating sites and present as multiple alleles.

**Table 3:** Tajima’s relative rate test of CBL proteins

Configuration	Phylogenetically distance sequences		
	MdCBL3, CsCBL3, PerCBL3	MeCBL3, BrCBL2-2, PtCBL3	FvCBL10-1, BrCBL2-2, MgCBL5
Identical sites in all three sequences	210	194	60
Divergent sites in all three sequences	1	4	60
Unique differences in Sequence A	1	4	36
Unique differences in Sequence B	10	18	16
Unique differences in Sequence C	1	2	23
*P-*value	0.00666	0.00284	0.00555
*X* ^*2*^ -test	7.36	8.91	7.69

Tajima’s relative rate test was carried out by randomly comparing three phylogenetically distant sequences of CBL proteins. The test was replicated for three times with one degree of freedom. In all the four cases, statistical result was found to be significant. The *P*-value less than 0.05 is often used to reject the null hypothesis of equal rates between lineages. Each analysis involved 3 amino acid sequences. All positions containing gaps and missing data were eliminated

**Table 4:** Tajima’s test for neutrality of OsCBL genes

*m*	*S*	*p*_s_	*Θ*	*π*	*D*
327	153	1.000000	0.157093	0.385669	4.413697

The analysis involved 327 amino acid sequences. All positions with less than 95 % site coverage were eliminated. That is, fewer than 5 % alignment gaps, missing data, and ambiguous bases were allowed at any position. There were a total of 153 positions in the final dataset. Evolutionary analyses were conducted in MEGA6. *Abbreviations*: *m* = number of sequences, *n* = total number of sites, *S* = Number of segregating sites, *p*_s_ = *S*/*n*, *Θ* = *p*_s_/a_1_, *π* = nucleotide diversity, and *D* is the Tajima test statistic

### Differential expression of OsCBL genes

**Fig. 7:**
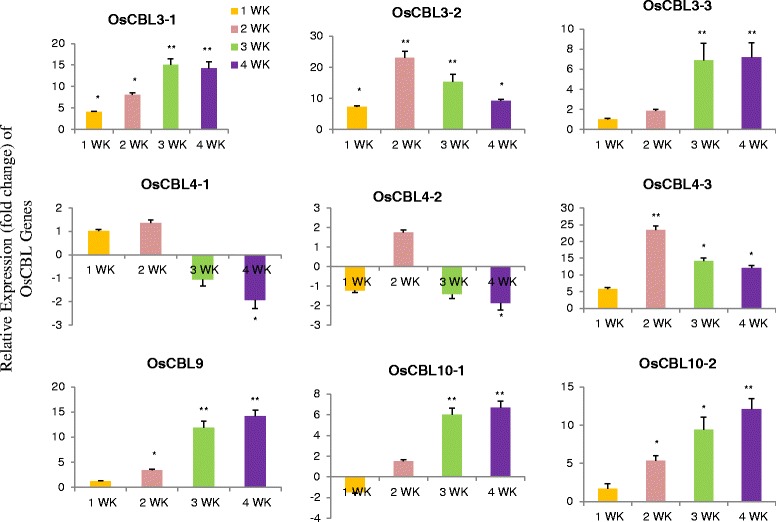
Time course quantitative gene expression of OsCBL genes at different developmental stages in *Oryza sativa*. The relative expression of OsCBL3-1, OsCBL3-2, OsCBL3-3, OsCBL9, and OsCBL10-2 genes are upregulated when compared to control gene and OsCBL4-1 and OsCBL4-2 are down regulated at 3rd and 4th week time point. Metric bar represents the standard error (SE). Asterisks indicate significant differences: *p < 0.05, **p < 0.01

**Fig. 8:**
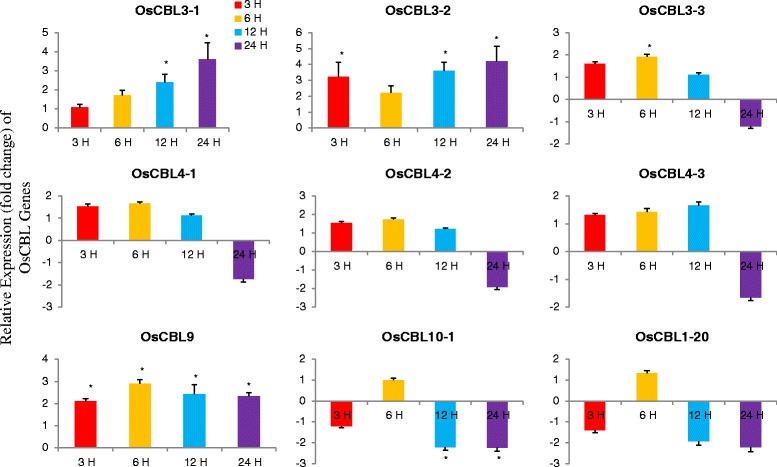
Time course quantitative gene expression of OsCBL genes in *Oryza sativa* treated with cold stress. The relative expression of OsCBL3-1, OsCBL3-2, and OsCBL9 genes undergoes up-regulation at all four time points. Expression of OsCBL3-3, OsCBL4-1, OsCBL4-2, OsCBL4-3, OsCBL10-1, and OsCBL10-2 undergoes down regulation at 4th week time point. Metric bar represents the standard error (SE). Asterisks indicate significant differences: *p < 0.05, **p < 0.01

**Fig. 9:**
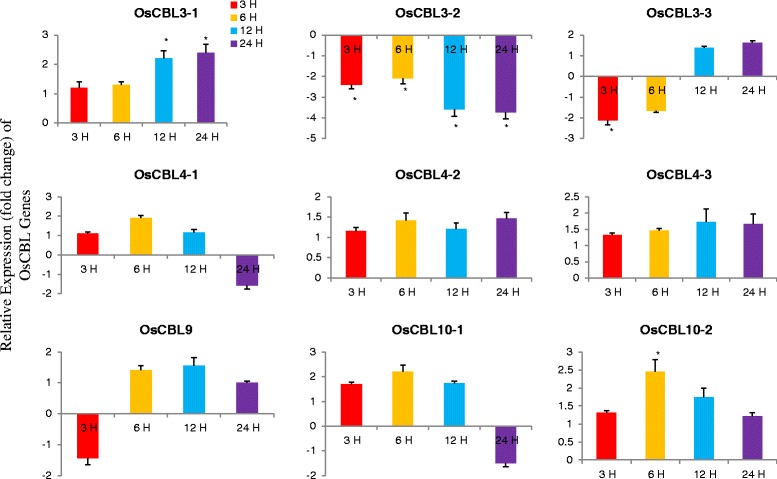
Time course quantitative gene expression of OsCBL genes in *Oryza sativa* treated with heat stress. The relative expression of OsCBL3-1, OsCBL4-2, OsCBL4-3, and OsCBL10-2 undergoes up-regulation at all four time points. The relative expression of OsCBL3-2 undergoes down regulation at all four time points. Metric bar represents the standard error (SE). Asterisks indicate significant differences: *p < 0.05, **p < 0.01

The plant have become the important target for genetic manipulation and provided an excellent platform for the investigation of different biological processes that control development. Analysis of these developmental processes at the molecular level requires isolation and characterization of important regulatory genes, including those are differentially expressed. Genes expressed in different developmental stages and specific tissues are of great interest. One of the major interests is whether the specific expression pattern of a gene in a specific cell or tissue type at a specific developmental stage can be used as a marker to study the development. Therefore, we investigated the relative expression patterns of the OsCBL gene at different developmental stages (Fig. 7). The relative expression of OsCBL genes in leaf tissue shows that OsCBL3-1, OsCBL3-2, OsCBL3-3, OsCBL4-3, OsCBL9 and OsCBL10-2 were upregulated at all four time points (Fig. 7). The expression of OsCBL4-1 undergone down regulation at the third and fourth week, while OsCBL4-2 undergone down regulation at weeks 1, 3 and 4. The major changes in the expression of the OsCBL genes were observed at 3 and 4 week. To better understand the role of CBL genes in stress responses, we conducted differential expression analysis of OsCBL genes by subjecting them to cold and heat stress at different time points (Fig. 8). The relative expression of OsCBL3-1, OsCBL3-2, and OsCBL9 was increased at all time points, whereas the expression of OsCBL4-1, OsCBL4-2, OsCBL4-3, OsCBL10-1 and OsCBL10-2 was down regulated at 24 h (Fig. 8). In heat treated plants, OsCBL3-1, OsCBL4-2, OsCBL4-3, and OsCBL10-2 had undergone up-regulation at all four time points (Fig. 9). The expression of OsCBL3-2 was down regulated at all the four time points (Fig. 9). The expression of OsCBL3-3 was down regulated at 3 and 6 h, and then gradually up-regulated at 12 and 24 h. Similarly, expression of OsCBL9 was down regulated at 3 h, but was gradually upregulated at 6, 12 and 24 h. Based on these findings, CBL genes are cold and heat stress responsive and differentially expressed upon exposure to different stresses.

## Conclusions

This study revealed that the basic architecture of CBL genes are conserved among all plant species, including green algae, bryophytes, pteridophytes, gymnosperms and angiosperms. The CBL genes of lower eukaryotes such as green algae and pinus appear to have evolved independently. Based on these findings, the split between chlorophyta (green algae) and embryophyta (higher plants) played an important role in the evolution of CBL genes. During the course of evolution, CBL signaling events by land plants expanded significantly via gene duplication. Expression analysis shows that OsCBL3-1, OsBL3-2, OsCBL3-3 and OsCBL10-2 significantly modulated during different developmental stages in *O. sativa*. The differential expression of OsCBL3-1 was significantly modulated during cold and heat stress suggesting its important roles during these events.

## Methods

The calcineurin B-like (CBL) gene family of *Arabidopsis thaliana* and *Oryza sativa* was downloaded from the *Arabidopsis* Information Resource (TAIR) database and the TIGR rice genome annotation project, respectively. Identified protein sequences of *A. thaliana* and *O. sativa* were then used to identify CBL gene family members in other plant species in the phytozome and spruce genome database [57]. The BLASTP program (default) was used to identify the CBL gene family members of other plant species. The default statistical parameters used in the BLASTP analysis were as follows: BLASTP-protein query of the protein database; expected threshold (E): −1, comparison matrix: BLOSUM62; no. of alignments to show: 100. Sequences having an E-value up to 7.0e-200 were taken into consideration for further analysis to cover the maximum number of genes. Collected protein sequences were then subjected to the scan prosite software to analyze the presence of EF-hand domains. Sequences having only three calcium binding EF-hands were as considered calcineurin B-like genes because all CBL proteins of *A. thaliana* contain only three calcium binding EF-hands. These CBL proteins were again subjected to BLASTP analysis against the *Arabidopsis* genome database using the default parameters to reconfirm them as CBL proteins. Sequences of plant species that gave BLASTP hits against the *A. thaliana* CBL proteins were considered as CBL proteins. The CBL genes were numbered according to the *Arabidopsis* CBL gene they matched during BLAST search to ensure proper orthologous numbering.

### Multiple sequence alignment and molecular modeling of CBL

The multiple sequence alignment of CBL genes of all species was carried out using the multalin software (http://multalin.toulouse.inra.fr/multalin/). The statistical parameters used to run the programs were as follows: protein weight matrix: BLOSSUM62, gap penalty at opening: default, gap penalty at extension: default, gap penalty at extremities: none, one iteration only: no, high consensus value: 90 % and low consensus value: 50 %.

### Construction of phylogenetic tree

To construct a phylogenetic tree, a clustal file was generated in the CLUSTALW software using the protein sequences of all CBL genes [58]. The parameters used to run the CLUSTALW program were as follows: protein weight matrix: BLOSSUM62, gap open: 10, gap extension: 0.2, iteration: none. The generated clustal file was downloaded and converted to MEGA file format using the MEGA5 software [52]. The generated MEGA file was then run in the MEGA5 software to construct the phylogenetic tree. The statistical parameters used to construct the phylogenetic tree were as follows: analysis: phylogenetic reconstruction, statistical method: maximum likelihood, test of phylogeny: bootstrap method, no. of bootstrap replicates: 3000, substitution type: amino acids, model/methods: Jones-Taylor-Thornton (JTT) model, rates among sites: uniform rates, gaps/missing data treatment: partial deletion, site coverage cutoff: 95 % and branch swap filter: very strong.

### Statistical analysis

Tajima’s relative rate test was carried out to investigate the significance and rate of evolution of plant CBL genes. The generated MEGA file used for the construction of the phylogenetic tree was subjected to MEGA5 to analyze Tajima’s relative rate test and Tajima’s test of neutrality. The statistical parameters used to run Tajima’s relative rate test were as follows: Tajima’s relative rate test scope: for three chosen sequences, substitution type: amino acid, and gaps/missing data treatment: complete deletion. The statistical parameters used to carry out Tajima’s test of neutrality were as follows: analysis: Tajima’s neutrality test, scope: all selected taxa, substitution type: amino acids, and gaps/missing data treatment: complete deletion.

### Plant treatment and quantitative real time PCR

Wild types *Oryza sativa* japonica var. nipponbare were grown in soil in a greenhouse under a16 h light: 8 h dark cycle at 22–25 °C for 20 days. Cold treatment consisted of 4 °C, while drought/heat treatment consisted of 40 °C. The leaves were sampled at 0, 3, 6, 12 and 24 h and immediately transferred to liquid nitrogen for subsequent analysis. Untreated plants were used as control samples. Three biological replicates were employed during this study. Total RNA was isolated from the treated and control samples using Trizol reagent. The RNA was quantified using Nanodrop1000 and its integrity was checked by electrophoresis in 1.5 % (w/v) agarose gel. High quality RNA was subjected to the preparation of cDNA using a Fermentas RevertAid first strand cDNA synthesis kit. The reactions were prepared by adding 1 μg total RNA, 2 μl of 10× RT buffer, 2 μl 10 mMdNTPs mix, 2 μl of oligo (dT)_18_ primer, 1 μl of reverse transcriptase, 1 μl ribolock RNase inhibitor and nuclease free sterile water up to 20 μl. The reaction mixtures were then subjected to thermal incubation at 42 °C for 60 min followed by reaction termination at 70 °C for 5 min. The generated cDNA was diluted 10 times and kept for further use. The primers of *O. sativa* CBL genes were designed using primer3 software targeting either the extreme 5′ end, which is not conserved, or the 3′ UTR region, which generated an amplicon size between 120 and 200 bp (primer length between 20 and 24 bp) (Additional file 4). The specificity of primers was checked through regular PCR amplification followed by agarose gel electrophoresis, as well as by the primer test in a Mx3000P quantitative real time PCR machine by examining the melting curve. The melting curve analysis of the primers was conducted at 60–95 °C, with a temperature increasing step of 0.06 °C/s (five acquisitions per degree of Celsius) at the end of each run. The quantitative real-time PCR was carried out using a Mx3000P real-time PCR system with SYBR green master mix (2x) (Fermentas) and ROX as a passive reference standard to normalize the SYBR fluorescent signal. The PCR amplification was carried out in a 25 μl reaction mixture containing 1 μl cDNA as the template, 12.5 μl SYBR green master mix (2X), 1 μl of each forward and reverse primer and nuclease free water up to 25 μl. The thermal profile for quantitative real time PCR was: initial activation at 95 °C for 10 min, followed by 40 cycles of 95 °C for 30 s, 60 °C for 30 s, and 72 °C for 30 s. Analyses were conducted in triplicate using three biological replicates. The primers showing efficiency of 90–105 % were considered as significant. The relative expression of OsCBL genes was calculated using 2^-ΔΔCt^ method [59].

## Additional files

Additional file 1:
**Table representing detailed genomic information of different CBL genes from 38 different plant species identified during this study.**
Additional file 2:
**Table representing molecular mass (kDa) and isoelectric point of different CBL genes from 38 plant species identified during this study.**
Additional file 3:
**Multiple sequence alignment of all CBL genes analyzed during this study.** Sequence alignment was done using online available Multalin software (http://multalin.toulouse.inra.fr/multalin/ multalin.html) using default programme. Multiple alignments show presence of different conserved domains and motifs in CBL genes. Additional file 4:
**Table showing lists of OsCBL primers used during qRT-PCR analysis.** Primers were designed using primer3 software (http://primer3.ut.ee/). Additional file 5:
**The details of phylogenetic datas are submitted to TreeBASE database and can be available in following link** http://purl.org/phylo/treebase/phylows/study/TB2:S17414?x-access-code=1b88565e08ce238f8fc7928d2fa11a12&format=html **.**

## Abbreviations

CBL: Calcineurin B-like
EF-hand: Elongation factor hand
CPK: Calcium dependent protein kinase
CaMs: Calmodulins
CIPK: CBL interacting protein kinase
