# Supplementary material for: Genome-wide identification of Calcineurin B-Like (CBL) gene family of plants reveals novel conserved motifs and evolutionary aspects in calcium signaling events
Source: BMC Plant Biol. 2015 Aug 6;15:189. doi: 10.1186/s12870-015-0543-0 (PMC4527274; doi:10.1186/s12870-015-0543-0)
Supplement: Additional file 1: — Table representing detailed genomic information of different CBL genes from 38 different plant species identified during this study. [file 12870_2015_543_MOESM1_ESM.pdf]

Additional Data File 1

Table representing genomic information of different CBL genes from 41 different plant species identified during this study.

Gene Name	Locus ID	ORF	No. of amino acids	No. of Introns	5'-3' Coordinate
Aguilegia coerulea					
AcCBL3	Aquca_002_00627	771	256	8	scaffold_2: 4944929 - 4951071
AcCBL4	Aquca_037_00212	648	215	8	scaffold_37: 1630325 - 1634926
AcCBL5	Aquca_037_00167	642	213	7	scaffold_37: 1337547 - 1339378
AcCBL9	Aquca_053_00098	642	513	8	scaffold_53: 1155128 - 1161725
AcCBL8	Aquca_015_00409	861	286	7	scaffold_15: 3971804 - 3976650
Arabidopsis thaliana					
AtCBL1	AT4G17615	639	213	7	9816452-9819312
AtCBL2	AT5G55990	678	226	7	22671452-22673855
AtCBL3	AT4G26570	690	230	8	13408422-13411070
AtCBL4	AT5G24270	666	222	8	8238534-8240366
AtCBL5	AT4G01420	609	203	7	580038-581518
AtCBL6	AT4G16350	678	226	7	9242320-9243912
AtCBL7	AT4G26560	642	214	6	13406733-13407973
AtCBL8	AT1G64480	642	214	7	23948028-23949751
AtCBL9	AT5G47100	639	213	8	19129640-19132283
AtCBL10	AT4G33000	768	256	8	15924349-15926628
Brachypodium distachyon					
BdCBL1	Bradi1g78640	639	212	8	Bd1: 74560611 - 74563115
BdCBL2-1	Bradi4g41910	672	223	8	Bd4: 45886683 - 45890617
BdCBL2-2	Bradi1g28160	672	223	8	Bd1: 23311516 - 23315385
BdCBL3	Bradi4g02740	678	225	7	Bd4: 2032401 - 2037220
BdCBL4-1	Bradi2g18740	660	219	7	Bd2: 16618956 - 16620835
BdCBL4-2	Bradi3g10640	642	213	8	Bd3: 8836542 - 8839301
BdCBL7	Bradi3g43510	669	222	7	Bd3: 45002221 - 45004557
BdCBL9	Bradi3g33700	645	214	7	Bd3: 36058940 - 36062627

BdCBL10	Bradi2g41730	969	322	8	Bd2: 42161056 - 42164141
Brassica rapa					
BrCBL1-1	Bra040169	642	213	7	A01: 4449274 - 4451276
BrCBL1-2	Bra012655	642	213	7	A03: 22897484 - 22899346
BrCBL2-1	Bra028949	663	220	6	A03: 5558818 - 5559993
BrCBL2-2	Bra035598	681	226	7	A02: 7361132 - 7362567
BrCBL3	Bra026421	657	218	7	A01: 9399149 - 9400482
BrCBL4-1	Bra009743	666	221	7	A06: 16967951 - 16969375
BrCBL4-2	Bra026462	666	221	7	A01: 9159114 - 9160610
BrCBL4-3	Bra029396	666	221	7	A02: 25418218 - 25419717
BrCBL8	Bra027703	645	214	7	A09: 6808374 - 6809938
BrCBL9-1	Bra022104	642	213	7	A02: 19404128 - 19405835
BrCBL9-2	Bra017504	642	213	7	A09: 15836083 - 15837869
BrCBL10-1	Bra034543	741	246	8	A08: 12911084 - 12912573
BrCBL10-2	Bra011404	636	211	7	A01: 2318789 - 2320114
BrCBL10-3	Bra037030	777	258	8	A03: 28829376 - 28831888
Capsella rubella					
CrCBL1	Carubv10005722m.g	642	213	8	scaffold_7: 9007671 - 9010450
CrCBL2	Carubv10027067m.g	681	226	9	scaffold_8: 8775616 - 8777856
CrCBL3	Carubv10005674m.g	681	226	8	scaffold_7: 5378153 - 5380856
CrCBL4	Carubv10001908m.g	669	222	8	scaffold_6: 8263830 - 8267997
CrCBL5	Carubv10003821m.g	618	205	7	scaffold_6: 16078670 - 16080252
CrCBL6	Carubv10005675m.g	681	226	7	scaffold_7: 9555984 - 9557691
CrCBL8	Carubv10022024m.g	645	214	7	scaffold_2: 113565 - 115256
CrCBL9	Carubv10027124m.g	642	213	8	scaffold_8: 224935 - 227681
CrCBL10	Carubv10006225m.g	726	241	7	scaffold_7: 2786048 - 2787603
Carica papaya					
CpCBL3	evm.TU.supercontig_12.107	627	208	6	supercontig_12: 968892 - 970793
CpCBL4	evm.TU.supercontig_3.83	642	213	7	supercontig_3: 678847 - 680422
CpCBL8	evm.TU.supercontig_571.2	651	216	7	supercontig_571: 7768 - 9855
CpCBL10	evm.TU.supercontig_7.168	744	247	7	supercontig_7: 1712780 - 1715218
Chlamydomonas reinhardtii					
CreinCBL8	Cre08.g363750	378	125	4	Chr8: 1296627 - 1300252
CreinCBL9	Cre16.g650750	768	255	5	Chr16: 1201218 - 1203629
Citrus clementina					

CcCBL1	Ciclev10022219m.g	642	213	8	scaffold_3: 4736047 - 4740432
CcCBL2	Ciclev10016575m.g	681	226	8	scaffold_2: 36331848 - 36338381
CcCBL3	Ciclev10009412m.g	672	223	9	scaffold_1: 23809096 - 23813097
CcCBL4	Ciclev10022148m.g	663	220	8	scaffold_3: 11418162 - 11423840
CcCBL8	Ciclev10023612m.g	645	214	7	scaffold_3: 833768 - 836097
CcCBL10-1	Ciclev10009236m.g	780	259	8	scaffold_1: 3862913 - 3866838
CcCBL10-2	Ciclev10026324m.g	768	255	8	scaffold_7: 6402025 - 6404869
Citrus sinensis					
CsCBL1	orange1.1g040252m.g	642	213	7	scaffold00095: 184907 - 188144
CsCBL2	orange1.1g027239m.g	681	226	8	scaffold00007: 2754231 - 2760324
CsCBL3	orange1.1g027336m.g	675	224	9	scaffold00070: 476357 - 480357
CsCBL4	orange1.1g027657m.g	663	220	7	scaffold00127: 334855 - 338286
CsCBL5	orange1.1g041619m.g	669	222	7	scaffold00009: 1628619 - 1630391
CsCBL8	orange1.1g048069m.g	642	213	6	scaffold00009: 1631555 - 1633444
CsCBL10-1	orange1.1g025162m.g	774	257	9	scaffold00005: 1583097 - 1587095
CsCBL10-2	orange1.1g025241m.g	768	255	8	scaffold00032: 975830 - 978497
Cuccumis sativus					
CsatCBL1	Cucsa.321410	642	213	8	scaffold03080: 1193694 - 1198025
CsatCBL2	Cucsa.044480	681	226	8	scaffold00542: 811864 - 816216
CsatCBL3	Cucsa.041730	672	223	9	scaffold00540: 295287 - 299109
CsatCBL4	Cucsa.032100	639	212	8	scaffold00429: 1511742 - 1516455
CsatCBL5	Cucsa.259240	651	216	7	scaffold02229: 5820805 - 582298
CsatCBL9	Cucsa.313710	563	186	6	scaffold02995: 2916027 - 2917644
CsatCBL10	Cucsa.395140	738	245	8	scaffold04100: 768320 - 771444
Eucalyptus grandis					
EgCBL1	Eucgr.D02136	672	223	8	scaffold_4: 35445421 - 35449583
EgCBL2	Eucgr.F03674	684	227	8	scaffold_6: 45063594 - 45068812
EgCBL3	Eucgr.E03829	600	199	8	scaffold_5: 67133025 - 67140510
EgCBL4-1	Eucgr.A01457	642	213	8	scaffold_1: 22973910 - 22978938
EgCBL4-2	Eucgr.K00375	642	213	8	scaffold_11: 4353773 - 4358630
EgCBL4-3	Eucgr.K00377	642	213	7	scaffold_11: 4404832 - 4406885
EgCBL4-4	Eucgr.K00380	675	224	7	scaffold_11: 4445843 - 4450929
EgCBL4-5	Eucgr.E00051	657	218	8	scaffold_5: 475094 - 479429
EgCBL8	Eucgr.D01757	645	214	8	scaffold_4: 31527514 - 31531067
EgCBL9	Eucgr.E00335	642	213	8	scaffold_5: 3147021 - 3151613

EgCBL10-1	Eucgr.F03125	747	248	8	scaffold_6: 40665506 - 40669113
EgCBL10-2	Eucgr.C00642	753	250	8	scaffold_3: 11661871 - 11665570
Fragaria vesca					
FvCBL3	gene01496-v1.0-hybrid	609	202	7	Chr3: 29314805 - 29316624
FvCBL4	gene12873-v1.0-hybrid	3048	1015	13	Chr7: 20612751 - 20620127
FvCBL8	gene32137-v1.0-hybrid	654	217	7	Chr5: 2970803 - 2972794
FvCBL9	gene10766-v1.0-hybrid	828	275	8	Chr5: 12503387 - 12514528
FvCBL10-1	gene23084-v1.0-hybrid	1128	375	10	Chr4: 23475187 - 23479811
FvCBL10-2	gene02679-v1.0-hybrid	924	307	8	Chr2: 18293344 - 18295103
Glycine max					
GmCBL1	Glyma17g15893	741	246	7	Chr17: 12617799 - 12621163
GmCBL2-1	Glyma08g44580	681	226	9	Chr8: 44155549 - 44160212
GmCBL2-2	Glyma18g08230	681	226	9	Chr18: 7009892 - 7014719
GmCBL3	Glyma07g39936	681	226	7	Chr7: 44285601 - 44290141
GmCBL4	Glyma06g13420	672	223	8	Chr6: 10562117 - 10569462
GmCBL5	Glyma08g02740	708	235	7	Chr8: 1881376 - 1884223
GmCBL9	Glyma05g05580	642	213	8	Chr5: 5016167 - 5025718
GmCBL10-1	Glyma08g20700	798	265	8	Chr8: 15711738 - 15715070
GmCBL10-2	Glyma17g34761	777	258	8	Chr17: 38736506 - 38741429
Gossipium raimondii					
GrCBL1-1	Gorai.003G178700	645	214	7	Chr3: 45019591 - 45023175
GrCBL1-2	Gorai.004G191400	630	209	8	Chr4: 50845719 - 50848597
GrCBL1-3	Gorai.007G030300	642	213	8	Chr7: 2078979 - 2081981
GrCBL1-4	Gorai.006G214700	666	221	8	Chr6: 46832994 - 46835660
GrCBL3-1	Gorai.013G150400	681	226	9	Chr13: 41048670 - 41051998
GrCBL3-2	Gorai.009G450400	681	226	9	Chr9: 70129486 - 70132973
GrCBL3-3	Gorai.002G102900	681	226	9	Chr2: 13114131 - 13117243
GrCBL4	Gorai.007G015400	663	220	8	Chr7: 1205668 - 1210620
GrCBL5	Gorai.008G255800	642	213	7	Chr8: 53819363 - 53821498
GrCBL8-1	Gorai.008G255900	648	215	8	Chr8: 53823934 - 53825963
GrCBL8-2	Gorai.006G207100	714	237	7	Chr6: 46249688 - 46251744
GrCBL10-1	Gorai.010G101400	759	252	8	Chr10: 18307537 - 18310406
GrCBL10-2	Gorai.009G045600	789	262	7	Chr9: 3290175 - 3292999
Linum usitatissimum					
LuCBL1	Lus10011028.g	642	213	6	scaffold1035: 189021 - 190213

LuCBL3	Lus10038764.g	627	208	6	scaffold34: 186908 - 188766
LuCBL4-1	Lus10018108.g	642	213	7	scaffold112: 114512 - 116060
LuCBL4-2	Lus10022407.g	642	213	7	scaffold38: 359573 - 361041
LuCBL4-3	Lus10030252.g	645	214	6	scaffold217: 435344 - 436506
LuCBL4-4	Lus10004006.g	585	194	6	scaffold1155: 49415 - 50699
LuCBL5	Lus10023069.g	645	214	6	scaffold325: 260473 - 261750
LuCBL8-1	Lus10001816.g	645	214	6	scaffold3494: 27077 - 28249
LuCBL8-2	Lus10003191.g	645	214	6	scaffold1056: 11857 - 13031
LuCBL9	Lus10032400.g	1182	393	11	scaffold291: 1126093 - 1129271
LuCBL10-1	Lus10015630.g	750	249	7	scaffold630: 143219 - 145200
LuCBL10-2	Lus10037648.g	753	250	7	scaffold196: 891538 - 893513
Malus domestica					
MdCBL1-1	MDP0000207134	642	213	7	MDC018264.217: 935 - 3960
MdCBL1-2	MDP0000262916	2142	713	16	MDC000196.715: 16866 - 25047
MdCBL2	MDP0000119547	1251	416	11	MDC001230.339: 611 - 3919
MdCBL3	MDP0000137075	1251	416	11	MDC007862.188: 1458 - 4766
MdCBL4-1	MDP0000155124	852	283	7	MDC021487.443: 12637 - 14656
MdCBL4-2	MDP0000774066	426	141	3	MDC011832.262: 2315 - 3059
MdCBL4-3	MDP0000263313	1098	365	8	MDC019137.162: 13946 - 2432
MdCBL5	MDP0000294682	426	141	3	MDC011832.250: 186 - 930
MdCBL8	MDP0000256696	1062	353	8	MDC001154.98: 1285 - 6384
MdCBL9	MDP0000259502	642	213	7	MDC016351.139: 1102 - 4127
MdCBL10	MDP0000186655	1302	433	9	MDC009688.195: 2014 - 4766
Manihot esculenta					
MeCBL1	cassava4.1_016071m.g	642	213	8	scaffold00738: 18389 - 24372
MeCBL3	cassava4.1_023888m.g	672	223	7	scaffold04443: 4953 - 6844
MeCBL4-1	cassava4.1_015878m.g	657	218	8	scaffold06916: 1363191 - 1366792
MeCBL4-2	cassava4.1_022392m.g	675	224	8	scaffold11495: 40001 - 42275
MeCBL5	cassava4.1_029089m.g	639	212	7	scaffold10243: 70731 - 72084
MeCBL8	cassava4.1_023193m.g	552	183	6	scaffold03175: 249972 - 252580
MeCBL9	cassava4.1_016083m.g	642	213	8	scaffold07290: 317824 - 324390
MeCBL10-1	cassava4.1_014701m.g	750	249	8	scaffold12118: 98181 - 100727
MeCBL10-2	cassava4.1_014733m.g	747	248	8	scaffold12525: 681612 - 684279
Medicago truncatula					
MtCBL2-1	AC233669_22	684	227	7	AC233669.2: 87017 - 91830

MtCBL2-2	Medtr2g027480	690	229	7	chr2: 8936060 - 8939186
MtCBL3-1	Medtr5g096420	681	226	10	chr5: 41120501 - 41124331
MtCBL3-2	Medtr3g060730	567	188	6	chr3: 18717150 - 18719337
MtCBL3-3	Medtr2g027440	690	229	7	chr2: 8914147 - 8917274
MtCBL3-4	Medtr2g027520	717	238	7	chr2: 8956964 - 8959758
MtCBL3-5	Medtr2g027500	675	224	7	chr2: 8946775 - 8949103
MtCBL4-1	Medtr3g091440	729	242	7	chr3: 31128431 - 31129967
MtCBL4-2	AC235758_37	927	308	8	AC235758.1: 197802 - 200175
MtCBL9	Medtr4g113510	1209	402	8	chr4: 38788169 - 38790502
MtCBL10	Medtr1g016430	882	293	9	chr1: 4762536 - 4767863
Micromonas pusila CCMP1545					
MpCBL1	MicpuC2.EuGene.0000130349	1116	371	2	scaffold_13: 690779 - 692089
MpCBL2	MicpuC2.EuGene.0000040369	1977	658	0	scaffold_4: 755423 - 757494
MpCBL6	e_gw1.15.503.1	582	193	1	scaffold_15: 578023 - 578753
Mimulus guttatus					
MgCBL1	mgv1a024164m.g	642	213	7	scaffold_409: 6885 - 8582
MgCBL3-1	mgv1a013241m.g	681	226	7	scaffold_4: 1669771 - 1672828
MgCBL3-2	mgv1a013314m.g	675	224	9	scaffold_24: 1003040 - 1007024
MgCBL4	mgv1a013679m.g	642	213	9	scaffold_51: 1342768 - 1345899
MgCBL5	mgv1a023934m.g	636	211	7	scaffold_158: 30663 - 32095
MgCBL8	mgv1a013491m.g	660	219	7	scaffold_60: 381340 - 383128
MgCBL9	mgv1a022772m.g	615	204	7	scaffold_242: 302834 - 304511
MgCBL10-2	mgv1a019977m.g	747	249	6	scaffold_36: 1602980 - 1604880
MgCBL10-1	mgv1a012532m.g	744	247	8	scaffold_79: 639017 - 64158
Oryza sativa					
OsCBL3-1	LOC_Os03g42840	678	226	7	23888729-23895481
OsCBL3-2	LOC_Os12g06510	885	295	7	3149519-3145028
OsCBL3-3	LOC_Os12g40510	678	226	7	25063167-25068213
OsCBL4-1	LOC_Os02g18880	642	214	7	11017808-11015828
OsCBL4-2	LOC_Os02g18930	642	214	7	11059975-11057896
OsCBL4-3	LOC_Os05g45810	633	211	7	26535878-26537911
OsCBL7	LOC_Os02g27940	519	173	5	16530749-16534064
OsCBL9	LOC_Os10g41510	642	214	7	22298890-22308687
OsCBL10-1	LOC_Os01g39770	873	291	8	22427831-22430870
OsCBL10-2	LOC_Os01g51420	801	267	8	29568547-29571484

Panicum halii					
PhCBL2	Pahal.0007s0427	678	225	7	scaffold_7: 3264721 - 3270895
PhCBL3	Pahal.0128s0167	672	223	8	scaffold_128: 1013195 - 1016588
PhCBL4-1	Pahal.0071s0163	708	235	8	scaffold_71: 1164918 - 1170629
PhCBL4-2	Pahal.0012s0184	582	193	7	scaffold_12: 1128865 - 1132251
PhCBL8	Pahal.0417s0014	636	211	7	scaffold_417: 59806 - 61904
PhCBL9	Pahal.0019s0042	642	213	7	scaffold_19: 578485 - 581870
PhCBL10-1	Pahal.0306s0048	798	265	9	scaffold_306: 281408 - 286795
PhCBL10-2	Pahal.0026s0062	897	298	8	scaffold_26: 948129 - 951472
Panicum virgatum					
PvCBL1	Pavirv00026000m.g	582	193	6	sg0.contig24310: 5436 - 7636
PvCBL3-1	Pavirv00044173m.g	678	225	7	sg0.contig37639: 451 - 6628
PvCBL3-2	Pavirv00040240m.g	672	223	8	sg0.contig59330: 733 - 4644
PvCBL3-3	Pavirv00003030m.g	672	223	7	sg0.contig34391: 1217 - 4915
PvCBL4-1	Pavirv00047304m.g	408	135	4	sg0.contig158895: 301 - 1904
PvCBL4-2	Pavirv00048053m.g	630	209	7	sg0.contig15791: 1725 - 4118
PvCBL4-3	Pavirv00017512m.g	657	218	7	sg0.contig49280: 1041 - 4453
PvCBL9	Pavirv00036730m.g	486	161	5	sg0.contig90606: 1043 - 3126
PvCBL10-1	Pavirv00069048m.g	798	265	8	sg0.contig08688: 8368 - 12268
PvCBL10-2	Pavirv00016899m.g	993	330	8	sg0.contig64375: 583 - 3718
Phaseolus vulgaris					
PvulCBL1	Phvul.002G002300	429	142	4	Chr2: 224863 - 226115
PvulCBL3-1	Phvul.003G088500	681	226	8	Chr3: 15697770 - 15704517
PvulCBL3-2	Phvul.008G086100	681	226	9	Chr8: 8534323 - 8538138
PvulCBL3-3	Phvul.006G135900	684	227	8	Chr6: 25018836 - 25023773
PvulCBL4	Phvul.009G052700	672	223	8	Chr9: 9855861 - 9859550
PvulCBL5	Phvul.002G299300	726	241	7	Chr2: 46173199 - 46175907
PvulCBL8	Phvul.002G299400	645	214	7	Chr2: 46177528 - 46179492
PvulCBL9	Phvul.003G225900	642	213	8	Chr3: 44590384 - 44594563
PvulCBL10-1	Phvul.010G139700	795	264	8	Chr10: 41180802 - 41184172
PvulCBL10-2	Phvul.001G034600	810	269	8	Chr1: 3357230 - 3362370
Physcomitrella patens					
PpCBL3-1	Phpat.005G037800	720	239	8	Chr5: 7291852 - 7295437
PpCBL3-2	Phpat.001G148800	642	213	7	Chr1: 25612965 - 25615986
PpCBL3-3	Phpat.013G011100	699	232	0	Chr13: 2094661 - 2096643

PpCBL9	Phpat.017G068000	642	213	6	Chr17: 12296563 - 12298892
Picea abies					
PaCBL1	MA_9965p0020	480	159	1	MA_9965:39941-47951
PaCBL2	MA_27386p0010	594	197	0	MA_27386:16112-16705
PaCBL3	MA_10431324p0020	525	174	0	MA_10434324:56856-57680
PaCBL4	MA_105065p0010	654	217	0	MA_105065:2006-2659
PaCBL5	MA_10204459p0010	579	192	0	MA_10204459:6350-6928
PaCBL6	MA_269415p0010	399	132	0	MA_269415:3966-4364
PaCBL7	MA_3268p0010	579	192	0	MA_3268:18919-1949
PaCBL8	MA_7799814p0010	516	171	0	MA_7799814:1771-2286
PaCBL9	MA_9445p0010	444	147	1	MA_9445:977-1736
PaCBL10	MA_18112p0010	342	113	0	MA_18112:6878-7219
PaCBL11	MA_10280648p0010	594	197	0	MA_10280648:580-1173
PaCBL12	MA_184231p0010	525	174	4	MA_184231:9354-15664
PaCBL13	MA_10288665p0010	543	180	4	MA_10288665:402-3876
Populous trichocarpa					
PtCBL2-1	Potri.006G002900	681	226	8	Chr6: 222523 - 228053
PtCBL2-2	Potri.016G003500	681	226	8	Chr16: 171318 - 176866
PtCBL2-3	Potri.001G371700	672	223	10	Chr1: 38550944 - 38556166
PtCBL3	Potri.011G094900	669	222	9	Chr11: 11534974 - 11539542
PtCBL4-1	Potri.015G013100	687	228	8	Chr15: 860437 - 864167
PtCBL4-2	Potri.012G015100	642	213	7	Chr12: 1474900 - 1476846
PtCBL4-3	Potri.015G013200	642	213	8	Chr15: 866349 - 869461
PtCBL5	Potri.003G141400	660	219	7	Chr3: 15838161 - 15840489
PtCBL8	Potri.001G090200	651	216	7	Chr1: 7094386 - 7096906
PtCBL9	Potri.001G150200	642	213	8	Chr1: 12367539 - 12372425
PtCBL10	Potri.006G230200	792	263	8	Chr6: 24076211 - 24079859
Prunus persica					
PperCBL1	ppa011375m.g	642	213	8	scaffold_5: 5812953 - 5818460
PperCBL3	ppa011040m.g	681	226	9	scaffold_6: 2982813 - 2986512
PperCBL4-1	ppa011404m.g	639	212	7	scaffold_2: 25773519 - 25775397
PperCBL4-2	ppa016322m.g	411	137	4	scaffold_2: 19299095 - 19300417
PperCBL5	ppa014557m.g	642	213	7	scaffold_5: 12141714 - 12143278
PperCBL8	ppa020531m.g	657	218	7	scaffold_5: 12144895 - 12147489
PperCBL10	ppa010315m.g	765	254	8	scaffold_1: 35023768 - 35026136

Ricinus communis					
RcCBL2	29836.t000025	675	224	7	29836: 333418 - 337938
RcCBL3	27893.t000011	672	223	7	27893: 103409 - 106423
RcCBL4-1	30024.t000035	657	218	7	30024: 709151 - 711555
RcCBL4-2	29742.t000053	639	212	7	29742: 328954 - 331610
RcCBL5	30190.t000089	633	210	7	30190: 3726876 - 3728694
RcCBL8	30190.t000090	732	243	7	30190: 3730456 - 3733274
RcCBL9	29822.t000180	432	143	5	29822: 1077872 - 1080485
RcCBL10	29794.t000028	750	249	8	29794: 147471 - 149591
Selaginella moellendorffii					
SmCBL2	409467	696	231	6	scaffold_10: 2234510 - 2235642
SmCBL3	272115	681	226	7	scaffold_105: 146851 - 148101
SmCBL5	81220	651	216	0	scaffold_4: 1393790 - 1394440
SmCBL9	131200	639	212	7	scaffold_105: 196957 - 198242
Setaria italica					
SiCBL3-1	Si037393m.g	678	225	7	scaffold_9: 11050067 - 11058738
SiCBL3-2	Si011013m.g	669	222	8	scaffold_7: 33686165 - 33691097
SiCBL3-3	Si023163m.g	678	225	7	scaffold_3: 47858858 - 47863509
SiCBL4-1	Si002829m.g	669	222	8	scaffold_5: 27353422 - 27356974
SiCBL4-2	Si023239m.g	636	211	7	scaffold_3: 12252958 - 12254800
SiCBL10-1	Si004544m.g	741	246	7	scaffold_5: 34928395 - 34931545
SiCBL10-2	Si002269m.g	960	319	8	scaffold_5: 32189444 - 32192113
Solanum lycopersicum					
SlCBL1-1	Solyc06g060980	648	215	7	Chr6: 35346305 - 35348757
SlCBL1-2	Solyc08g007160	648	215	8	Chr8: 1710704 - 1715488
SlCBL1-3	Solyc08g077770	642	213	8	Chr8: 58838680 - 58845331
SlCBL3-1	Solyc07g065820	660	219	7	Chr7: 64652286 - 64656394
SlCBL3-2	Solyc12g015870	675	224	7	Chr12: 5860247 - 5866106
SlCBL3-3	Solyc02g032310	660	219	0	Chr2: 18911726 - 18912385
SlCBL4-1	Solyc06g051970	645	214	8	Chr6: 32132164 - 32135700
SlCBL4-2	Solyc03g083320	645	214	8	Chr3: 46697415 - 46700140
SlCBL8-1	Solyc00g034810	690	229	7	Chr0: 12856697 - 12858684
SlCBL8-2	Solyc12g055920	681	226	8	Chr12: 47273184 - 47276237
SlCBL10	Solyc08g065330	774	257	8	Chr8: 50446717 - 50455805
Solanum tuberosum					

StCBL1-1	PGSC0003DMG400026580	648	215	7	Chr6: 40567033 - 40569526
StCBL1-2	PGSC0003DMG400020493	786	261	6	Chr8: 1062935 - 1068924
StCBL1-3	PGSC0003DMG400004554	642	213	7	Chr8: 37531845 - 37538586
StCBL3-1	PGSC0003DMG400014370	669	222	7	Chr10: 540001 - 545887
StCBL3-2	PGSC0003DMG400022267	660	219	7	Chr7: 52352875 - 52358455
StCBL3-3	PGSC0003DMG400038238	675	224	0	Chr2: 40324722 - 40325396
StCBL3-4	PGSC0003DMG400029807	675	224	7	Chr12: 9143132 - 9152065
StCBL4-1	PGSC0003DMG400019601	645	214	7	Chr6: 33740222 - 33743628
StCBL4-2	PGSC0003DMG401009127	645	214	7	Chr3: 17240489 - 17244626
StCBL5	PGSC0003DMG400005840	360	119	3	Chr8: 5001212 - 5002749
StCBL8	PGSC0003DMG400017886	588	195	6	Chr3: 1896804 - 1898593
StCBL10	PGSC0003DMG400029942	873	290	9	Chr8: 24305080 - 24311104
Sorghum bicolor					
SbCBL3-1	Sobic.008G046500	672	223	8	Chr8: 4551042 - 4555624
SbCBL3-2	Sobic.008G152800	1107	368	8	Chr8: 51114830 - 51120709
SbCBL4-1	Sobic.009G210300	639	212	7	Chr9: 55666215 - 55668914
SbCBL4-2	Sobic.004G130600	642	213	8	Chr4: 16910354 - 16913556
SbCBL4-3	Sobic.003G208400	657	218	9	Chr3: 53980001 - 53985782
SbCBL9	Sobic.001G294300	642	213	7	Chr1: 50108965 - 50113190
SbCBL10-1	Sobic.003G196400	960	319	8	Chr3: 52183173 - 52187695
SbCBL10-2	Sobic.003G275000	852	283	9	Chr3: 61146592 - 61150090
Thelluginella halophila					
ThCBL1	Thhalv10026227m.g	642	213	8	scaffold_1: 9974443 - 9977290
ThCBL2	Thhalv10014594m.g	681	226	8	scaffold_2: 10141529 - 10143383
ThCBL3	Thhalv10026170m.g	681	226	9	scaffold_1: 5727528 - 5730228
ThCBL4	Thhalv10004891m.g	663	220	7	scaffold_6: 3122807 - 3124323
ThCBL6	Thhalv10027149m.g	687	228	7	scaffold_1: 10765835 - 10767111
ThCBL8	Thhalv10023688m.g	645	214	8	scaffold_8: 159635 - 162402
ThCBL9	Thhalv10001023m.g	642	213	7	scaffold_20: 2228708 - 2230894
ThCBL10-1	Thhalv10026019m.g	780	259	8	scaffold_1: 2934341 - 2936275
ThCBL10-2	Thhalv10028908m.g	756	251	8	scaffold_3: 7161432 - 7163434
Theobroma cacao					
TcCBL1	Thecc1EG015835	642	213	8	scaffold_3: 29895701 - 29899471
TcCBL2	Thecc1EG030465	681	226	8	scaffold_6: 27160570 - 27165634
TcCBL3	Thecc1EG030580	681	226	10	scaffold_7: 402592 - 407103

TcCBL4	Thecc1EG015411	642	213	8	scaffold_3: 27815301 - 27820121
TcCBL5	Thecc1EG016487	660	219	7	scaffold_3: 33079096 - 33081102
TcCBL8	Thecc1EG016488	645	214	8	scaffold_3: 33081831 - 33086215
TcCBL10	Thecc1EG037279	765	254	8	scaffold_9: 2698544 - 2701954
Vitis venifera					
VvCBL1-1	GSVIVG01004840001	642	213	8	Chr2: 5573344 - 5579529
VvCBL1-2	GSVIVG01004842001	642	213	8	Chr2: 5592160 - 5598339
VvCBL3-1	GSVIVG01004678001	555	184	6	Chr Un: 39588970 - 39592698
VvCBL3-2	GSVIVG01014744001	681	226	8	Chr 19: 9146520 - 9157750
VvCBL4	GSVIVG01038549001	642	213	9	Chr16: 21954571 - 21960272
VvCBL5	GSVIVG01019554001	792	263	8	Chr2: 1576333 - 1578897
VvCBL8	GSVIVG01019555001	660	219	8	Chr2: 1579135 - 1583057
VvCBL10-1	GSVIVG01035369001	777	258	8	Chr4: 833252 - 838822
VvCBL10-2	GSVIVG01035370001	756	251	8	Chr4: 841053 - 846927
Zea mays					
ZmCBL3-1	GRMZM2G173424	678	225	8	Chr3: 115357901 - 115384056
ZmCBL3-2	GRMZM2G033680	672	223	8	Chr10: 7865133 - 7869500
ZmCBL3-3	GRMZM2G010093	672	223	8	Chr10: 7727567 - 7731272
ZmCBL4-1	GRMZM2G001221	636	211	7	Chr6: 159259837 - 159262707
ZmCBL4-2	GRMZM2G137751	645	214	6	Chr2: 29393210 - 29394827
ZmCBL4-3	GRMZM2G110080	645	214	6	Chr4: 220332704 - 220334495
ZmCBL9-1	GRMZM2G015324	642	213	7	Chr9: 114457085 - 114462156
ZmCBL9-2	GRMZM2G107575	642	213	7	Chr1: 94985068 - 94989733
ZmCBL10	GRMZM2G116584	963	320	8	Chr3: 220520716 - 220524593

Supplementary Table 2

Supplementary able representing molecular mass (in kDa) and isoelectric point of different CBL genes from 41 plant species identified during this study

Gene Name	Locus ID	Mol. Weight (kD)	pI
AcCBL3	Aquca_002_00627	29.620	5.14
AcCBL4	Aquca_037_00212	24.475	4.83
AcCBL5	Aquca_037_00167	24.559	4.70
AcCBL9	Aquca_053_00098	24.455	4.86
AcCBL10	Aquca_015_00409	33.001	5.58
Arabidopsis thaliana			
AtCBL1	AT4G17615	24.553	4.48
AtCBL2	AT5G55990	25.809	4.65
AtCBL3	AT4G26570	26.537	4.55
AtCBL4	AT5G24270	25.693	4.87
AtCBL5	AT4G01420	23.517	4.56
AtCBL6	AT4G16350	26.028	5.65
AtCBL7	AT4G26560	24.404	4.53
AtCBL8	AT1G64480	24.649	4.76
AtCBL9	AT5G47100	24.531	4.32
AtCBL10	AT4G33000	29.342	4.46
Brachypodium distachyon			
BdCBL1	Bradi1g78640	24.412	4.83
BdCBL2-1	Bradi4g41910	25.742	4.96
BdCBL2-2	Bradi1g28160	25.728	4.96
BdCBL3	Bradi4g02740	25.767	4.98
BdCBL4-1	Bradi2g18740	24.704	5.10
BdCBL4-2	Bradi3g10640	24.358	4.95
BdCBL7	Bradi3g43510	25.485	4.82
BdCBL9	Bradi3g33700	24.446	4.89

BdCBL10	Bradi2g41730	36.409	5.91
Brassica rapa			
BrCBL1-1	Bra040169	24.586	4.82
BrCBL1-2	Bra012655	24.603	4.77
BrCBL2-1	Bra028949	25.151	5.11
BrCBL2-2	Bra035598	25.865	5.04
BrCBL3	Bra026421	24.885	4.96
BrCBL4-1	Bra009743	25.582	5.14
BrCBL4-2	Bra026462	25.482	5.00
BrCBL4-3	Bra029396	25.369	4.87
BrCBL8	Bra027703	24.751	5.37
BrCBL9-1	Bra022104	24.350	4.75
BrCBL9-2	Bra017504	24.350	4.75
BrCBL10-1	Bra034543	28.462	4.95
BrCBL10-2	Bra011404	24.335	4.77
BrCBL10-3	Bra037030	29.874	4.70
Capsella rubella			
CrCBL1	Carubv10005722m.g	24.481	4.87
CrCBL2	Carubv10027067m.g	25.843	5.04
CrCBL3	Carubv10005674m.g	26.031	4.93
CrCBL4	Carubv10001908m.g	25.653	5.14
CrCBL5	Carubv10003821m.g	23.881	5.24
CrCBL6	Carubv10005675m.g	26.007	5.50
CrCBL8	Carubv10022024m.g	24.678	5.17
CrCBL9	Carubv10027124m.g	24.496	4.80
CrCBL10	Carubv10006225m.g	27.689	4.79
Carica papaya			
CpCBL3	evm.TU.supercontig_12.107	24.071	5.03
CpCBL4	evm.TU.supercontig_3.83	24.358	4.93
CpCBL8	evm.TU.supercontig_571.2	24.858	5.18
CpCBL10	evm.TU.supercontig_7.168	28.907	5.16
Chlamydomonas reinhardtii			
CreinCBL8	Cre08.g363750	14.291	4.23
CreinCBL9	Cre16.g650750	27.418	4.67
Citrus clementina			

CcCBL1	Ciclev10022219m.g	24.435	4.81
CcCBL2	Ciclev10016575m.g	26.043	4.84
CcCBL3	Ciclev10009412m.g	25.668	4.93
CcCBL4	Ciclev10022148m.g	25.110	4.87
CcCBL8	Ciclev10023612m.g	24.442	5.01
CcCBL10-1	Ciclev10009236m.g	29.685	4.85
CcCBL10-2	Ciclev10026324m.g	28.954	4.66
Citrus sinensis			
CsCBL1	orange1.1g040252m.g	24.421	4.81
CsCBL2	orange1.1g027239m.g	26.057	4.84
CsCBL3	orange1.1g027336m.g	25.815	4.93
CsCBL4	orange1.1g027657m.g	25.110	4.87
CsCBL5	orange1.1g041619m.g	25.360	5.11
CsCBL8	orange1.1g048069m.g	24.315	4.87
CsCBL10-1	orange1.1g025162m.g	29.410	4.71
CsCBL10-2	orange1.1g025241m.g	29.000	4.71
Cucumis sativus			
CsatCBL1	Cucsa.321410	24.533	4.69
CsatCBL2	Cucsa.044480	25.655	4.87
CsatCBL3	Cucsa.041730	25.882	4.88
CsatCBL4	Cucsa.032100	24.530	4.84
CsatCBL5	Cucsa.259240	24.667	5.64
CsatCBL9	Cucsa.313710	21.460	4.72
CsatCBL10	Cucsa.395140	28.318	4.85
Eucalyptus grandis			
EgCBL1	Eucgr.D02136	25.387	5.00
EgCBL2	Eucgr.F03674	26.062	4.83
EgCBL3	Eucgr.E03829	23.003	4.95
EgCBL4-1	Eucgr.A01457	24.323	4.86
EgCBL4-2	Eucgr.K00375	24.483	4.92
EgCBL4-3	Eucgr.K00377	24.255	4.96
EgCBL4-4	Eucgr.K00380	25.761	5.28
EgCBL4-5	Eucgr.E00051	24.656	4.91
EgCBL8	Eucgr.D01757	24.655	4.89
EgCBL9	Eucgr.E00335	24.403	4.92

EgCBL10-1	Eucgr.F03125	28.032	5.51
EgCBL10-2	Eucgr.C00642	29.032	4.85
Fragaria vesca			
FvCBL3	gene01496-v1.0-hybrid	23.389	5.14
FvCBL4	gene12873-v1.0-hybrid	115.266	5.97
FvCBL8	gene32137-v1.0-hybrid	24.980	5.01
FvCBL9	gene10766-v1.0-hybrid	31.855	6.81
FvCBL10-1	gene23084-v1.0-hybrid	42.436	5.85
FvCBL10-2	gene02679-v1.0-hybrid	35.693	5.20
Glycine max			
GmCBL1	Glyma17g15893	28.256	5.12
GmCBL2-1	Glyma08g44580	26.037	4.83
GmCBL2-2	Glyma18g08230	26.065	4.88
GmCBL3	Glyma07g39936	25.777	4.78
GmCBL4	Glyma06g13420	25.543	4.79
GmCBL5	Glyma08g02740	26.725	4.90
GmCBL9	Glyma05g05580	24.416	4.87
GmCBL10-1	Glyma08g20700	30.778	5.14
GmCBL10-2	Glyma17g34761	29.255	4.84
Gossipium raimondii			
GrCBL1-1	Gorai.003G178700	24.450	4.84
GrCBL1-2	Gorai.004G191400	23.862	4.81
GrCBL1-3	Gorai.007G030300	24.375	4.86
GrCBL1-4	Gorai.006G214700	25.392	5.10
GrCBL3-1	Gorai.013G150400	25.961	4.94
GrCBL3-2	Gorai.009G450400	25.982	4.93
GrCBL3-3	Gorai.002G102900	25.882	4.98
GrCBL4	Gorai.007G015400	24.914	4.87
GrCBL5	Gorai.008G255800	24.581	4.80
GrCBL8-1	Gorai.008G255900	27.260	5.29
GrCBL8-2	Gorai.006G207100	24.482	5.15
GrCBL10-1	Gorai.010G101400	29.255	4.86
GrCBL10-2	Gorai.009G045600	30.312	5.07
Linum usitatissimum			
LuCBL1	Lus10011028.g	24.425	5.01

LuCBL3	Lus10038764.g	23.771	5.02
LuCBL4-1	Lus10018108.g	24.629	4.87
LuCBL4-2	Lus10022407.g	24.532	4.92
LuCBL4-3	Lus10030252.g	24.795	4.93
LuCBL4-4	Lus10004006.g	22.316	4.83
LuCBL5	Lus10023069.g	45.258	5.27
LuCBL8-1	Lus10001816.g	24.732	5.48
LuCBL8-2	Lus10003191.g	24.891	5.17
LuCBL9	Lus10032400.g	20.348	5.64
LuCBL10-1	Lus10015630.g	28.316	4.78
LuCBL10-2	Lus10037648.g	28.275	4.83
Malus domestica			
MdCBL1-1	MDP0000207134	24.416	4.87
MdCBL1-2	MDP0000262916	79.456	9.40
MdCBL2	MDP0000119547	47.470	7.94
MdCBL3	MDP0000137075	47.470	7.94
MdCBL4-1	MDP0000155124	32.594	5.77
MdCBL4-2	MDP0000774066	16.236	4.62
MdCBL4-3	MDP0000263313	16.385	4.82
MdCBL5	MDP0000294682	41.463	7.77
MdCBL8	MDP0000256696	39.019	6.24
MdCBL9	MDP0000259502	24.416	4.87
MdCBL10	MDP0000186655	49.290	8.30
Manihut esculenta			
MeCBL1	cassava4.1_016071m.g	24.559	4.75
MeCBL3	cassava4.1_023888m.g	25.836	4.88
MeCBL4-1	cassava4.1_015878m.g	25.360	5.31
MeCBL4-2	cassava4.1_022392m.g	25.697	4.71
MeCBL5	cassava4.1_029089m.g	24.326	4.64
MeCBL8	cassava4.1_023193m.g	20.977	4.72
MeCBL9	cassava4.1_016083m.g	24.497	4.87
MeCBL10-1	cassava4.1_014701m.g	28.741	4.82
MeCBL10-2	cassava4.1_014733m.g	28.559	4.83
Medicago truncatula			
MtCBL2-2	AC233669_22	25.972	4.90

MtCBL2-3	Medtr2g027480	26.307	4.95
MtCBL3-1	Medtr5g096420	26.064	4.83
MtCBL3-2	Medtr3g060730	21.695	4.90
MtCBL3-3	Medtr2g027440	26.171	4.79
MtCBL3-4	Medtr2g027520	27.082	4.88
MtCBL3-5	Medtr2g027500	25.682	4.79
MtCBL4-1	Medtr3g091440	27.814	5.29
MtCBL4-2	AC235758_37	35.072	5.24
MtCBL9	Medtr4g113510	46.431	9.61
MtCBL10	Medtr1g016430	33.099	4.91
Micromonas pusila			
MpCBL1	MicpuC2.EuGene.0000130349	25.972	4.90
MpCBL2	MicpuC2.EuGene.0000040369	70.365	9.51
MpCBL6	e_gw1.15.503.1	21.846	5.49
Mimulus guttatus			
MgCBL1	mgv1a024164m.g	24.458	4.80
MgCBL3-1	mgv1a013241m.g	25.969	4.83
MgCBL3-2	mgv1a013314m.g	25.800	4.94
MgCBL4	mgv1a013679m.g	24.647	4.87
MgCBL5	mgv1a023934m.g	24.434	4.86
MgCBL8	mgv1a013491m.g	24.916	4.64
MgCBL9	mgv1a022772m.g	23.474	4.74
MgCBL10-1	mgv1a012532m.g	28.329	4.85
MgCBL10-2	mgv1a019977m.g	28.718	5.13
Oryza sativa			
OsCBL3-1	LOC_Os03g42840	25.803	4.508
OsCBL3-2	LOC_Os12g06510	33.479	4.79
OsCBL3-3	LOC_Os12g40510	25.864	4.54
OsCBL4-1	LOC_Os02g18880	24.399	4.52
OsCBL4-2	LOC_Os02g18930	24.491	4.65
OsCBL4-3	LOC_Os05g45810	23.916	4.71
OsCBL7	LOC_Os02g27940	19.311	4.24
OsCBL9	LOC_Os10g41510	24.496	4.40
OsCBL10-1	LOC_Os01g39770	32.892	4.58
OsCBL10-2	LOC_Os01g51420	29.915	4.61

Panicum halii			
PhCBL2	Pahal.0007s0427	25.771	4.94
PhCBL3	Pahal.0128s0167	25.727	4.96
PhCBL4-1	Pahal.0071s0163	21.305	5.32
PhCBL4-2	Pahal.0012s0184	22.417	5.24
PhCBL8	Pahal.0417s0014	23.907	4.94
PhCBL9	Pahal.0019s0042	24.353	4.77
PhCBL10-1	Pahal.0306s0048	29.789	5.10
PhCBL10-2	Pahal.0026s0062	34.076	5.38
Panicum vigatum			
PvCBL1	Pavirv00026000m.g	22.180	5.05
PvCBL3-1	Pavirv00044173m.g	25.785	4.94
PvCBL3-2	Pavirv00040240m.g	25.727	4.96
PvCBL3-3	Pavirv00003030m.g	25.757	4.96
PvCBL4-1	Pavirv00047304m.g	15.510	6.70
PvCBL4-2	Pavirv00048053m.g	23.701	4.77
PvCBL4-3	Pavirv00017512m.g	24.856	4.98
PvCBL9	Pavirv00036730m.g	18.796	4.77
PvCBL10-1	Pavirv00069048m.g	29.858	4.85
PvCBL10-2	Pavirv00016899m.g	37.204	5.73
Phaseolus vulgaris			
PvulCBL1	Phvul.002G002300	16.549	4.83
PvulCBL3-1	Phvul.003G088500	25.846	4.88
PvulCBL3-2	Phvul.008G086100	26.071	4.83
PvulCBL3-3	Phvul.006G135900	26.096	5.25
PvulCBL4	Phvul.009G052700	25.666	4.84
PvulCBL5	Phvul.002G299300	27.641	5.01
PvulCBL8	Phvul.002G299400	24.534	5.11
PvulCBL9	Phvul.003G225900	24.472	4.94
PvulCBL10-1	Phvul.010G139700	30.157	5.00
PvulCBL10-2	Phvul.001G034600	30.123	4.92
Physcomitrella Patens			
PpCBL3-1	Phpat.005G037800	27.374	4.90
PpCBL3-2	Phpat.001G148800	24.538	5.18
PpCBL3-3	Phpat.013G011100	26.628	4.91

PpCBL9	Phpat.017G068000	24.527	5.17
Picea abies			
PaCBL1	MA_9965p0020	17.798	5.18
PaCBL2	MA_27386p0010	22.260	4.40
PaCBL3	MA_10431324p0020	20.018	4.33
PaCBL4	MA_105065p0010	23.282	6.41
PaCBL5	MA_10204459p0010	21.223	4.54
PaCBL6	MA_269415p0010	14.687	4.40
PaCBL7	MA_3268p0010	21.272	4.36
PaCBL8	MA_7799814p0010	19.455	4.23
PaCBL9	MA_9445p0010	16.084	4.37
PaCBL10	MA_18112p0010	12.774	4.02
PaCBL11	MA_10280648p0010	21.632	4.54
PaCBL12	MA_184231p0010	20.126	5.42
PaCBL13	MA_10288665p0010	20.988	5.32
Populous trichocarpa			
PtCBL2-1	Potri.006G002900	25.887	4.90
PtCBL2-2	Potri.016G003500	25.894	4.84
PtCBL2-3	Potri.001G371700	25.672	4.93
PtCBL3	Potri.011G094900	25.769	4.88
PtCBL4-1	Potri.015G013100	26.014	5.07
PtCBL4-2	Potri.012G015100	24.498	4.88
PtCBL4-3	Potri.015G013200	24.009	4.78
PtCBL5	Potri.003G141400	24.994	4.70
PtCBL8	Potri.001G090200	24.660	4.77
PtCBL9	Potri.001G150200	24.340	4.79
PtCBL10	Potri.006G230200	30.234	4.96
Prunus persica			
PperCBL1	ppa011375m.g	24.542	4.87
PperCBL3	ppa011040m.g	26.052	4.94
PperCBL4-1	ppa011404m.g	24.475	4.88
PperCBL4-2	ppa016322m.g	16.091	4.52
PperCBL5	ppa014557m.g	24.820	4.82
PperCBL8	ppa020531m.g	25.206	5.05
PperCBL10	ppa010315m.g	29.509	4.74

Ricinus communis			
RcCBL2	29836.t000025	25.731	4.81
RcCBL3	27893.t000011	25.735	4.83
RcCBL4-1	30024.t000035	25.466	5.00
RcCBL4-2	29742.t000053	24.125	4.81
RcCBL5	30190.t000089	24.252	4.59
RcCBL8	30190.t000090	27.787	4.93
RcCBL9	29822.t000180	16.537	4.63
RcCBL10	29794.t000028	28.760	4.87
Selaginella moellendorffii			
SmCBL2	409467	26.721	4.99
SmCBL3	272115	26.182	5.29
SmCBL5	81220	24.311	5.34
SmCBL9	131200	24.314	4.80
Setaria italica			
SiCBL3-1	Si037393m.g	25.833	4.93
SiCBL3-2	Si011013m.g	25.614	4.96
SiCBL3-3	Si023163m.g	25.884	4.99
SiCBL4-1	Si002829m.g	25.256	4.99
SiCBL4-2	Si023239m.g	23.703	4.93
SiCBL10-1	Si004544m.g	27.857	5.44
SiCBL10-2	Si002269m.g	36.581	5.58
Solanum lycopersicum			
SlCBL1-1	Solyc06g060980.1	24.557	4.79
SlCBL1-2	Solyc08g007160.2	24.645	4.79
SlCBL1-3	Solyc08g077770.2	24.295	5.00
SlCBL3-1	Solyc07g065820.2	25.179	4.87
SlCBL3-2	Solyc12g015870.1	25.682	4.88
SlCBL3-3	Solyc02g032310.1	25.396	4.94
SlCBL4-1	Solyc06g051970.2	24.591	4.64
SlCBL4-2	Solyc03g083320.2	24.571	4.64
SlCBL8-1	Solyc00g034810.1	26.767	6.02
SlCBL8-2	Solyc12g055920.1	26.663	6.23
SlCBL10	Solyc08g065330.2	29.877	4.77
Solanum tuberosum			

StCBL1-1	PGSC0003DMG400026580	24.472	4.71
StCBL1-2	PGSC0003DMG400020493	30.356	5.80
StCBL1-3	PGSC0003DMG400004554	24.322	5.00
StCBL3-1	PGSC0003DMG400014370	25.515	4.99
StCBL3-2	PGSC0003DMG400022267	25.223	4.87
StCBL3-3	PGSC0003DMG400038238	25.903	4.79
StCBL3-4	PGSC0003DMG400029807	25.682	4.88
StCBL4-1	PGSC0003DMG400019601	24.618	4.76
StCBL4-2	PGSC0003DMG401009127	24.628	4.64
StCBL5	PGSC0003DMG400005840	13.610	4.61
StCBL8	PGSC0003DMG400017886	22.649	5.48
StCBL10	PGSC0003DMG400029942	32.745	4.79
Sorghum bicolor			
SbCBL3-1	Sobic.008G046500	25.676	4.96
SbCBL3-2	Sobic.008G152800	41.244	7.94
SbCBL4-1	Sobic.009G210300	24.047	4.77
SbCBL4-2	Sobic.004G130600	24.386	4.83
SbCBL4-3	Sobic.003G208400	25.107	5.05
SbCBL9	Sobic.001G294300	24.485	4.89
SbCBL10-1	Sobic.003G196400	35.866	5.29
SbCBL10-2	Sobic.003G275000	32.289	5.47
Thelluginella halophila			
ThCBL1	Thhalv10026227m.g	24.570	4.82
ThCBL2	Thhalv10014594m.g	25.829	5.04
ThCBL3	Thhalv10026170m.g	25.907	4.87
ThCBL4	Thhalv10004891m.g	25.393	5.22
ThCBL6	Thhalv10027149m.g	26.378	5.27
ThCBL8	Thhalv10023688m.g	24.636	5.27
ThCBL9	Thhalv10001023m.g	24.384	4.75
ThCBL10-1	Thhalv10026019m.g	30.015	5.16
ThCBL10-2	Thhalv10028908m.g	28.948	4.81
Theobroma cacao			
TcCBL1	Thecc1EG015835	24.298	4.86
TcCBL2	Thecc1EG030465	25.880	4.84
TcCBL3	Thecc1EG030580	25.986	4.83

TcCBL4	Thecc1EG015411	24.351	5.05
TcCBL5	Thecc1EG016487	25.129	4.60
TcCBL8	Thecc1EG016488	24.652	4.90
TcCBL10	Thecc1EG037279	29.393	4.87
Vitis venifera			
VvCBL1-1	GSVIVG01004840001	24.472	4.81
VvCBL1-2	GSVIVG01004842001	24.472	4.81
VvCBL3-1	GSVIVG01004678001	21.261	4.86
VvCBL3-2	GSVIVG01014744001	26.009	4.93
VvCBL4	GSVIVG01038549001	24.518	4.82
VvCBL5	GSVIVG01019554001	30.093	5.07
VvCBL8	GSVIVG01019555001	25.244	4.82
VvCBL10-1	GSVIVG01035369001	29.812	5.15
VvCBL10-2	GSVIVG01035370001	28.987	5.00
Zea mays			
ZmCBL3-1	GRMZM2G173424	25.911	4.98
ZmCBL3-2	GRMZM2G033680	25.707	5.01
ZmCBL3-3	GRMZM2G010093	25.661	4.96
ZmCBL4-1	GRMZM2G001221	23.967	4.87
ZmCBL4-2	GRMZM2G137751	24.582	4.86
ZmCBL4-3	GRMZM2G110080	24.582	4.86
ZmCBL9-1	GRMZM2G015324	24.453	4.79
ZmCBL9-2	GRMZM2G107575	24.471	4.89
ZmCBL10	GRMZM2G116584	35.849	5.46

Supplementary Figure

Multiple sequence alignment of all CBL genes analyzed during this study. Sequence alignment was done using online available Multalin software (http://multalin.toulouse.inra.fr/multalin/ multalin.html) using default programme. Multiple alignments show presence of different conserved domains and motifs in CBL genes.

```
           361  370       380       390       400       410       420       430       440       450       460       470       480       490       500       510       520       530       540
           |--------+---------+---------+---------+---------+---------+---------+---------+---------+---------+---------+---------+---------+---------+---------+---------+---------+---------|
AcCBL9     MGCFHSK-----------GSK----QY-------
AtCBL1     MGCFHSK-----------AAK----EF-------
CrCBL1     MGCFHSK-----------AAR----EF-------
BrCBL1-1   MGCFHSK-----------VAR----EF-------
ThCBL1     MGCFHSK-----------VAR----EF-------
BrCBL1-2   MGCFQSK-----------VAR----EF-------
AtCBL9     MGCFHST-----------AAR----EF-------
CrCBL9     MGCFHST-----------AAR----EF-------
BrCBL9-1   MGCLHST-----------AAR----EF-------
BrCBL9-2   MGCLHST-----------AAR----EY-------
ThCBL9     MGCFHST-----------ASR----EF-------
CcCBL1     MGCFQSK-----------VAK----QF-------
CsCBL1     MGCFQSK-----------VAK----QF-------
VvCBL1-1   MGCFQST-----------ARK----QF-------
VvCBL1-2   MGCFQST-----------ARK----QF-------
MdCBL9     MGCFSSK-----------ARR----QF-------
MdCBL1-1   MGCFSSK-----------ARR----QF-------
PperCBL1   MGCFSSK-----------ARR----QF-------
CsatCBL1   MGCIQSK-----------GSR----QY-------
EgCBL9     MGCFHST-----------QRR----QF-------
PtCBL9     MGCFSSK-----------VPR----QF-------
GrCBL1-3   MGCFQSK-----------VTR----QY-------
TcCBL1     MGCFQSK-----------VTR----QY-------
SlCBL1-3   MGCFNSK-----------VKK----QF-------
StCBL1-3   MGCFNSK-----------VKK----QF-------
MeCBL1     MGCASSK-----------VAR----QF-------
MeCBL9     MGCFSSK-----------VAR----QF-------
LuCBL1     MGCFSSK-----------VIR----KF-------
MgCBL1     MGCFQST-----------SRK----QF-------
MgCBL9                         K----QF-------
BdCBL1     MGCFHSK-----------PRR----QY-------
BdCBL9     MGCIQSK-----------AKR---RQH-------
OsCBL9     MGCFQST-----------ARR----PR-------
SbCBL9     MGCFHST-----------AKR----QH-------
ZmCBL9-2   MGCFHST-----------AKR----QH-------
ZmCBL9-1   MGCFHST-----------AKR----QH-------
PhCBL9     MGCFAST-----------AKR----QH-------
SlCBL1-1   MGCFLSK-----------ATK----KF-------
StCBL1-1   MGCFHST-----------AAK----KF-------
EgCBL1     MGCLPSS-----------QRK----QC-------
GrCBL1-4   MGCLQCK-----------AKR----LC-------
GrCBL1-2   MSCFHSK-----------VTR-------------
GmCBL1     MVLMIYISVKFEYTGTVLCCLTCISSIMGCMNSK-----------SVR----KF-------
GmCBL9     MGCFHSK-----------SAR----QF-------
PvulCBL9   MGCFVSR-----------SRR----KF-------
PpCBL3-2   MGCFHSK-----------KNK----VP-------
SmCBL9     MGCFHSK-----------PSQ----HA-------
SlCBL1-2   MGCFSSK-----------VARG--HQF-------
StCBL1-2   MGCFSSK-----------VARG--HQF-------
FvCBL9     MSSLVNVEEREVSLATYAATFLQIVNQRRHQMDVQGNRPKRKFYSLLSPRWKLPQMVTTRTGV-----------CVPK--RKF-------
GrCBL1-1      MKGN-----------LLTL--CCF-------
PvCBL1     MGCFAST-----------AKR----QH-------
CsatCBL9
AcCBL4     MGCVYSK-----------KVR-RS----------
TcCBL4     MGCVSSK-----------RRK-QE----------
CcCBL4     MGCVLTK-----------KTK-QT----------
CsCBL4     MGCVLTK-----------KTK-QT----------
PtCBL4-1   MGCYLSK-----------RTK-KT----------
PtCBL4-2   MGCYHSK-----------RTK-ET----------
EgCBL4-1   MGCFCSK-----------GVK-RT----------
MgCBL4     MGCFHSK-----------ITK-HT----------
VvCBL4     MGCFCSK-----------KAR-QT----------
LuCBL4-1   MGCFQSK-----------TTK-LT----------
LuCBL4-2   MGCFQSK-----------TTK-QT----------
CsatCBL4   MGCYHSK-----------KPK--S----------
```

```
[illegible]
PperCBL4-1  MGCYWSK-----------KAK--T----------
SlCBL4-1    MGCFHSK-----------STLHYT----------
StCBL4-1    MGCFHSK-----------TTLHYT----------
SlCBL4-2    MGCFPSK-----------ICRFST----------
StCBL4-2    MGCFHSK-----------ICRFST----------
MeCBL4-1    MLVAFKRCFCSK-----------KNK-HT----------
RcCBL4-1    MLVAFNCCFCIK-----------RPK-QI----------
MeCBL4-2    MQAGFSSCFCLK-----------RSK-KT----------
CpCBL4      MGCISSK-----------TTK-YT----------
GrCBL4      MGCGSSK-----------RSK-QL----------
GmCBL4      MGCYCST-----------SKKTEA----------
PvulCBL4    MGCCYST-----------SKRTGT----------
EgCBL4-2    MGCTISK-----------KSE-RT----------
EgCBL4-3    MGCITSK-----------SFA-RT----------
EgCBL4-4    MGCIISK-----------NSE-QT----------
PtCBL4-3    MGCCQSK-----------GTK-TT----------
AtCBL4      MGCSVSK-----------KKKKNAMRP-------
CrCBL4      MGCSVSK-----------KKKKNTIRP-------
BrCBL4-1    MGCAPSK-----------KKT-NALRP-------
ThCBL4      MGCSPSK-----------RKK--ATRP-------
BrCBL4-2    MGCSLSK-----------KK--NAIRP-------
BrCBL4-3    MGCSLSK-----------KKK-IAIPP-------
BdCBL4-1    MGCGASS-----------ALK-RHKRA-------
OsCBL4-3    MGCASS--------------K-QFKRP-------
PvCBL4-2    MGCATSS-----------QFG-A---A-------
SiCBL4-2    MGCASSR-----------QFG-A-RAA-------
PhCBL8      MGCASSR-----------QFG-A-RAA-------
SbCBL4-1    MGCASSK-----------QFG-R-SAS-------
ZmCBL4-1    MGCATSK-----------QFS-R-SAP-------
BdCBL4-2    MGCASSK-----------QFK-R--AP-------
OsCBL4-1    MGCISSK-----------QFK-R--AA-------
OsCBL4-2    MGCVSSK-----------QFK-R--AA-------
SbCBL4-2    MGCVYSK-----------GSK-R---P-------
ZmCBL4-2    MGCVSSK-----------ESR-R--RP-------
ZmCBL4-3    MGCVSSK-----------ESR-R--RP-------
PhCBL4-2    MGCVSSK-----------QFK-R--AK-------
PvCBL4-3    MGCVPTK-----------HAG-RSPHS-------
SiCBL4-1    MGCIPTK-----------HAG-QSPDS-------
SbCBL4-3    MGCLQTK-----------HAS-RSPHL-------
AtCBL8      MLAFVKCFSLK-----------RAK----HP-------
CrCBL8      MLAFVKCFSLK-----------RTK----HP-------
ThCBL8      MLAFVKCFSLK-----------RTK----HP-------
BrCBL8      MLAFVKRFSLK-----------RTK----HP-------
CpCBL8      MSAFKSCFCMK-----------KYG----KR-------
PtCBL8      MDAFKGCFCST-----------KTS----KL-------
CcCBL8      MNAFGRCFCMK-----------KSK----QI-------
EgCBL8      MQSLFGCFCVR-----------KGQ----RK-------
TcCBL8      MRAFKACFCLK-----------KVR----QK-------
VvCBL8      MLAFTRCFCLK-----------KAK----QH-------
RcCBL8      MRALKGCFSLK-----------KSI----KQ-------
FvCBL8      MPPFMSCFCFK-----------SSRV-YRHS-------
PperCBL8    MRRFLHCFCLM-----------SSKV-DRHR-------
GrCBL8-2    MGCFCLK-----------KVR----NK-------
GrCBL8-1    MHTLMGCFCLK-----------KLR----HK-------
MgCBL8      MRSLVNFFCLS-----------KSK----RP-------
PvulCBL8    MSKSMRCFCLK-----------KAR----PK-------
LuCBL8-1    MLAFGRCFFKQ-----------KIK----KQ-------
LuCBL8-2    MLAFRHCFFKQ-----------KIK----KQ-------
EgCBL4-5    MGCGPSKRAKE-----------SATPEAKPS-------
LuCBL4-3    MGCTSSKTAAQ------------DEP------------
RcCBL4-2    MGCLPSKEAGP-----------VTAP------------
PpCBL9      MGCLHSK-----------HHK----LP-------
LuCBL5      MLAFRGCFSKQK-----------IKK-----Q-------
MdCBL8      MLHCFCFKSSR-----------VDR-----L-------
MtCBL4-2    MGCSSSKVAENIMDGSSEVGKDKNLPSQVKE-----------NEL-----I-------
MdCBL4-1    MPSYLDLLLTAKLFYSHSVLLIVQELRVNYVGLLMGCYCSKE-----------AKT-----P-------
MdCBL5      MEATRAERLPVNERDRHVCVFLGGESDGGMIDAREFATRILG-----------SVC-----Y-------
MtCBL4-1    MLKHLAMRIPQFLLLKHLVSIHPS-----------SSL-----T-------
AcCBL5      MGCVWTK-----------G-Q-------------
PperCBL5    MGCVWMK-----------Q-R-------------
VvCBL5      MILFSRLHQLADSIHSLDRTSPNFSFFNSESCVRYIVFSLYSLTSDAIPSQKKVFLMGCACMK-----------Q-R-------------
TcCBL5      MGCACVK-----------R-L-------------
CsCBL5      MGCVCMK-----------Q-R-------------
MeCBL5      MGCLCTK-----------Q-R-------------
RcCBL5      MGCLFTK-----------Q-Q-------------
PtCBL5      MLCSYNFLSSKEVYLLTLKSSF-MGCICTK-----------Q-Q-------------
GmCBL5      MGCCCTK-----------Q-R-------------
PvulCBL5    MGCCCTK-----------E-R-------------
MgCBL5      MGCVCTK-----------K-R-------------
SlCBL8-1    MWFFLVLNWIMHSLKGCFCLK-----------KSR-------------
SlCBL8-2    MWFFLVLNWIMHSLKGCFCLK-----------KSR-------------
StCBL8      MHSLKDCFCLK-----------KTR-------------
CsCBL8
MeCBL8
```

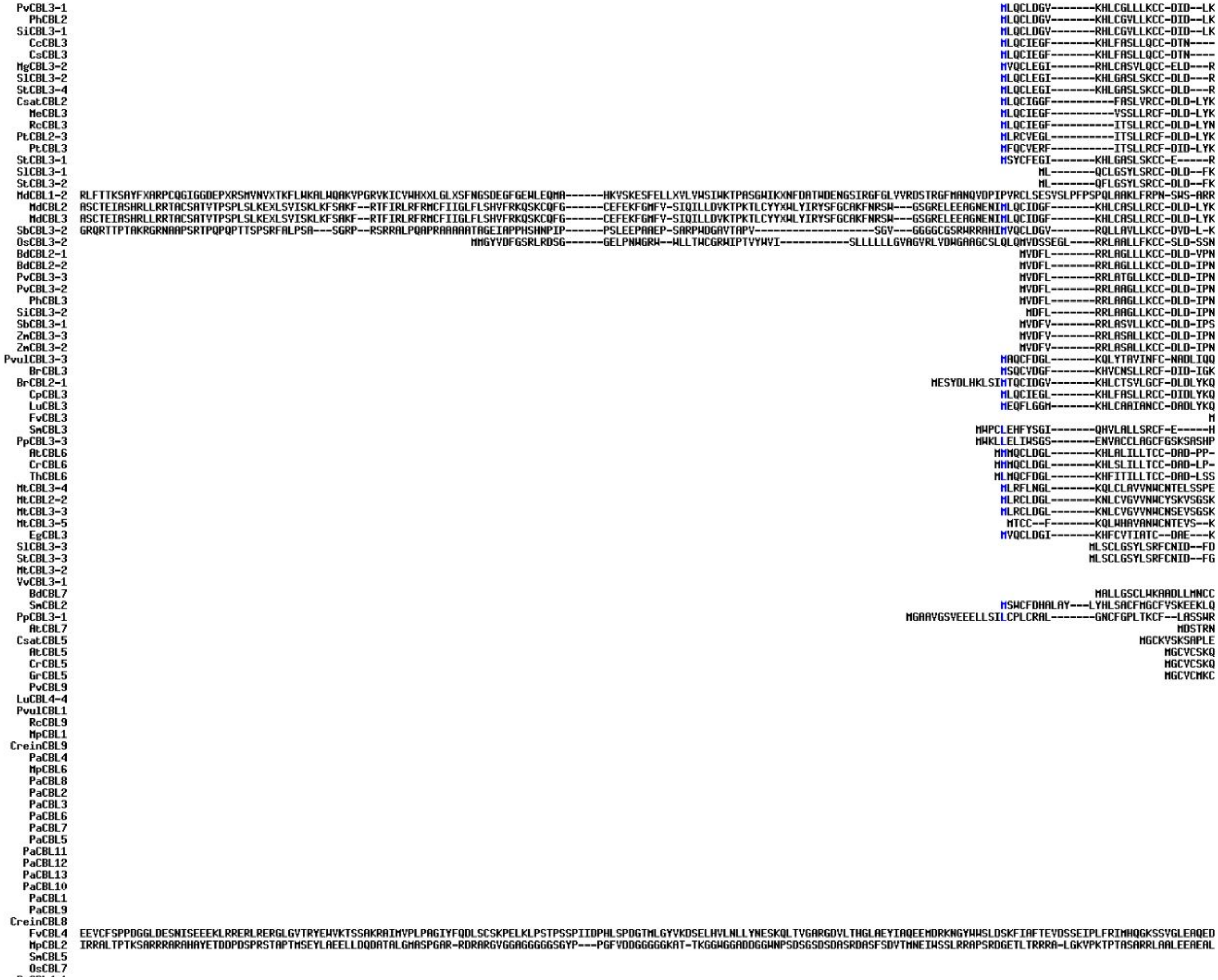
PvCBL3-1
PhCBL2
SiCBL3-1
CcCBL3
CsCBL3
MgCBL3-2
SlCBL3-2
StCBL3-4
CsatCBL2
MeCBL3
RcCBL3
PtCBL2-3
PtCBL3
StCBL3-1
SlCBL3-1
StCBL3-2
MdCBL1-2
MdCBL2
MdCBL3
SbCBL3-2
OsCBL3-2
BdCBL2-1
BdCBL2-2
PvCBL3-3
PvCBL3-2
PhCBL3
SiCBL3-2
SbCBL3-1
ZmCBL3-3
ZmCBL3-2
PvulCBL3-3
BrCBL3
BrCBL2-1
CpCBL3
LuCBL3
FvCBL3
SmCBL3
PpCBL3-3
AtCBL6
CrCBL6
ThCBL6
MtCBL3-4
MtCBL2-2
MtCBL3-3
MtCBL3-5
EgCBL3
SlCBL3-3
StCBL3-3
MtCBL3-2
VvCBL3-1
BdCBL7
SmCBL2
PpCBL3-1
AtCBL7
CsatCBL5
AtCBL5
CrCBL5
GrCBL5
PvCBL9
LuCBL4-4
PvulCBL1
RcCBL9
MpCBL1
CreinCBL9
PaCBL4
MpCBL6
PaCBL8
PaCBL2
PaCBL3
PaCBL6
PaCBL7
PaCBL5
PaCBL11
PaCBL12
PaCBL13
PaCBL10
PaCBL1
PaCBL9
CreinCBL8
FvCBL4
MpCBL2
SmCBL5
OsCBL7

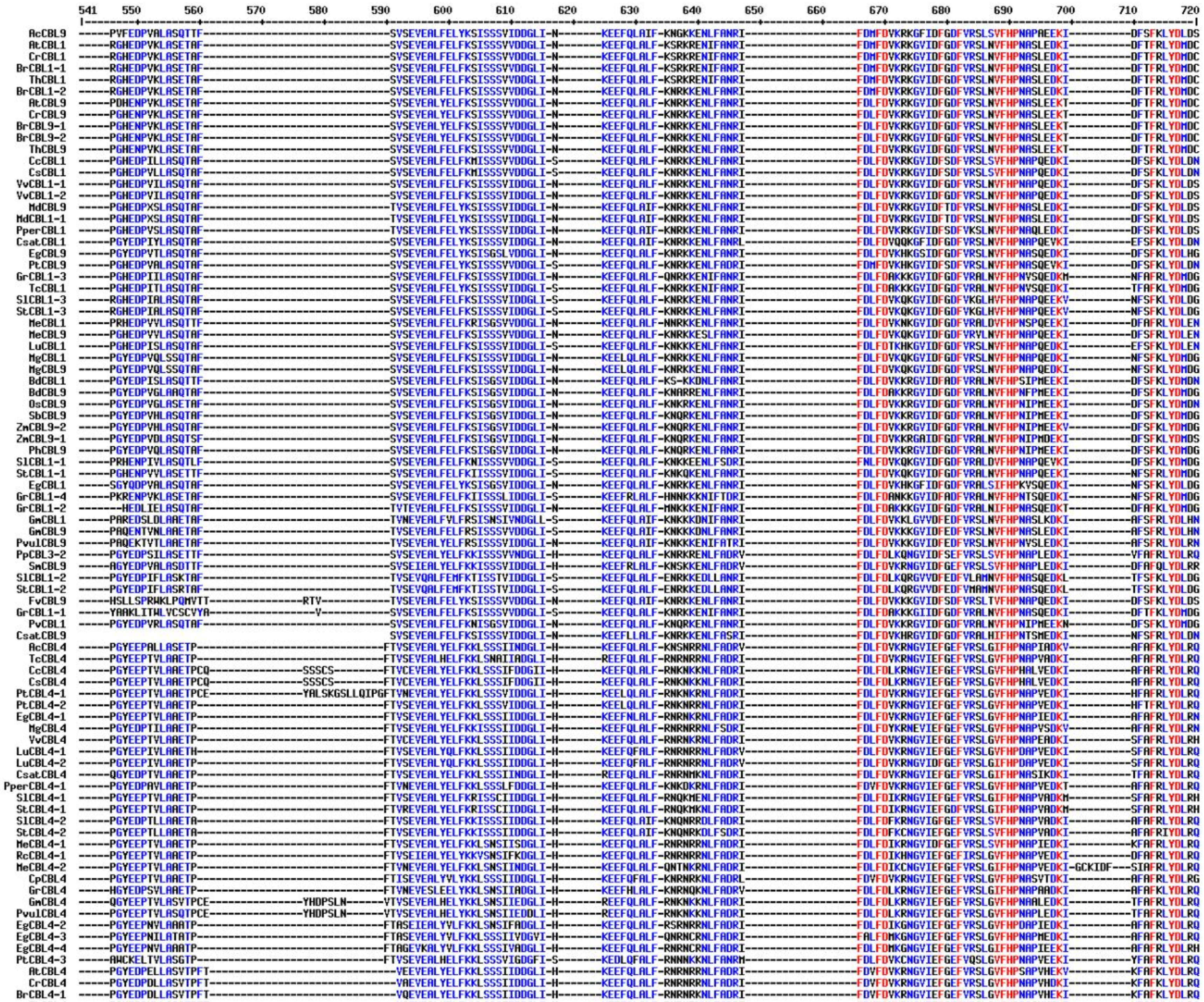

541 550 560 570 580 590 600 610 620 630 640 650 660 670 680 690 700 710 720
AcCBL9
AtCBL1
CrCBL1
BrCBL1-1
ThCBL1
BrCBL1-2
AtCBL9
CrCBL9
BrCBL9-1
BrCBL9-2
ThCBL9
CcCBL1
CsCBL1
VvCBL1-1
VvCBL1-2
MdCBL9
MdCBL1-1
PperCBL1
CsatCBL1
EgCBL9
PtCBL9
GrCBL1-3
TcCBL1
SlCBL1-3
StCBL1-3
MeCBL1
MeCBL9
LuCBL1
MgCBL1
MgCBL9
BdCBL1
BdCBL9
OsCBL9
SbCBL9
ZmCBL9-2
ZmCBL9-1
PhCBL9
SlCBL1-1
StCBL1-1
EgCBL1
GrCBL1-4
GrCBL1-2
GmCBL1
GmCBL9
PvulCBL9
PpCBL3-2
SmCBL9
SlCBL1-2
StCBL1-2
FvCBL9
GrCBL1-1
PvCBL1
CsatCBL9
AcCBL4
TcCBL4
CcCBL4
CsCBL4
PtCBL4-1
PtCBL4-2
EgCBL4-1
MgCBL4
VvCBL4
LuCBL4-1
LuCBL4-2
CsatCBL4
PperCBL4-1
SlCBL4-1
StCBL4-1
SlCBL4-2
StCBL4-2
MeCBL4-1
RcCBL4-1
MeCBL4-2
CpCBL4
GrCBL4
GmCBL4
PvulCBL4
EgCBL4-2
EgCBL4-3
EgCBL4-4
PtCBL4-3
AtCBL4
CrCBL4
BrCBL4-1

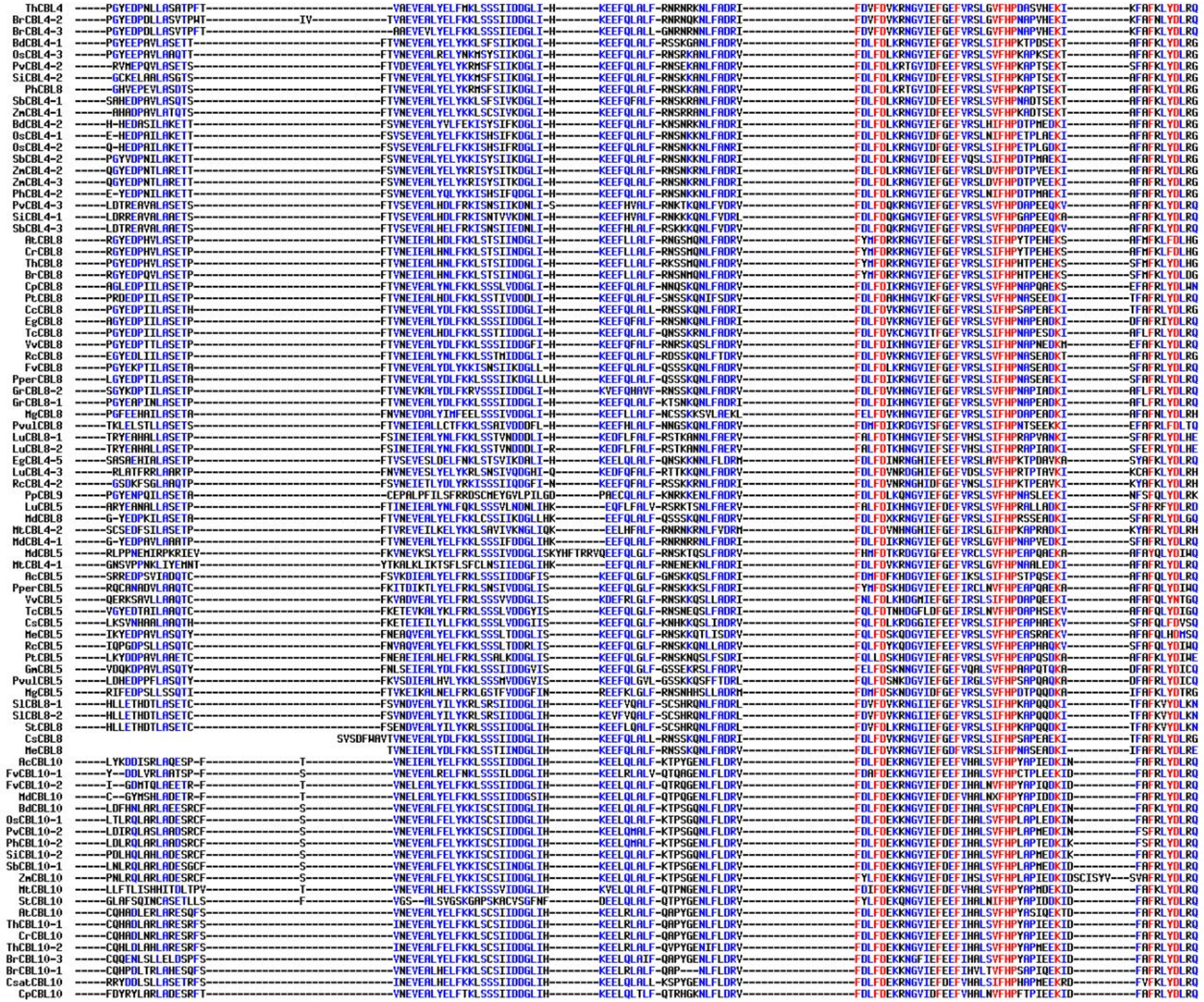

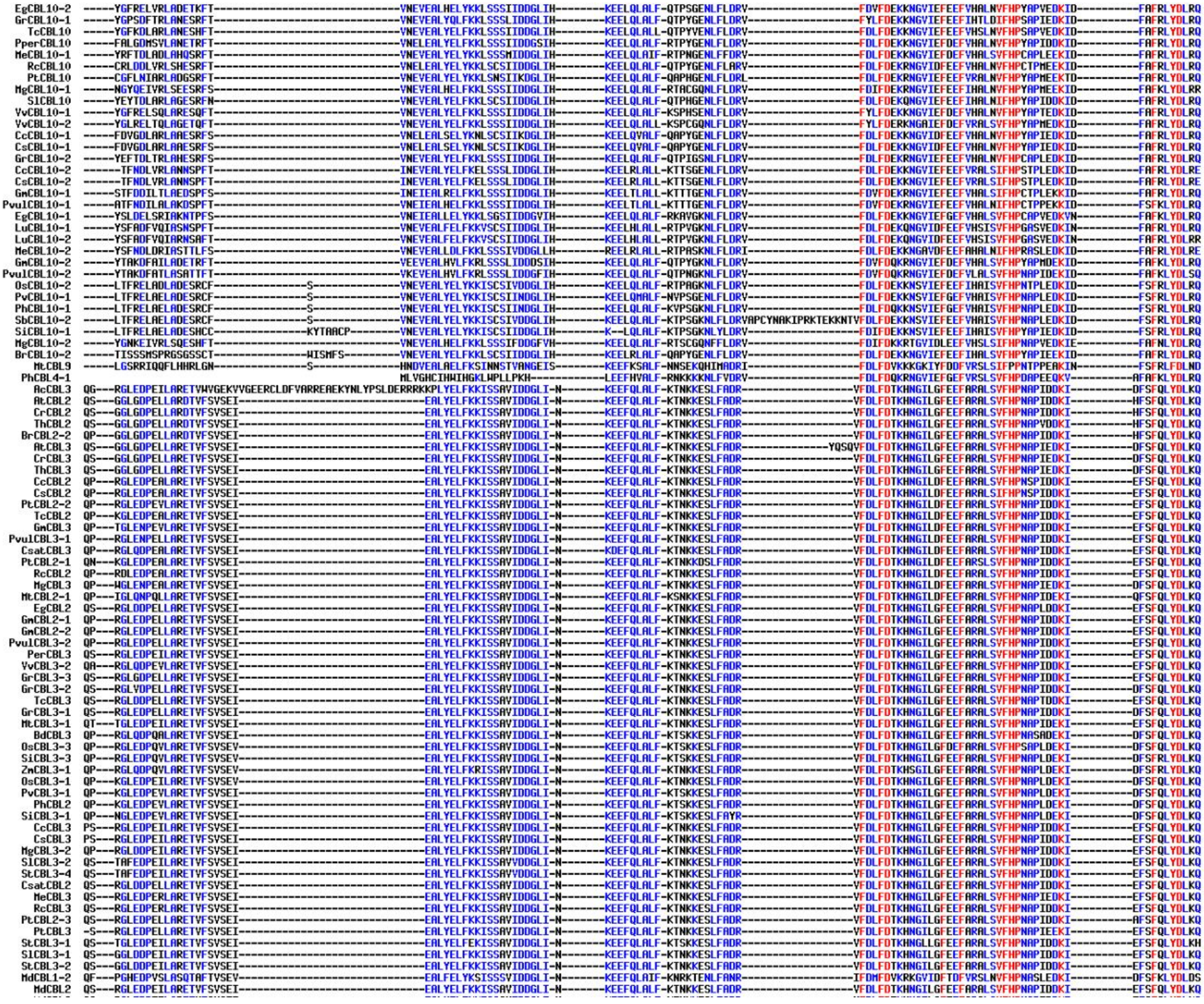
EgCBL10-2
GrCBL10-1
TcCBL10
PperCBL10
MeCBL10-1
RcCBL10
PtCBL10
MgCBL10-1
SlCBL10
VvCBL10-1
VvCBL10-2
CcCBL10-1
CsCBL10-1
GrCBL10-2
CcCBL10-2
CsCBL10-2
GmCBL10-1
PvulCBL10-1
EgCBL10-1
LuCBL10-1
LuCBL10-2
MeCBL10-2
GmCBL10-2
PvulCBL10-2
OsCBL10-2
PvCBL10-1
PhCBL10-1
SbCBL10-2
SiCBL10-1
MgCBL10-2
BrCBL10-2
MtCBL9
PhCBL4-1
AcCBL3
AtCBL2
CrCBL2
ThCBL2
BrCBL2-2
AtCBL3
CrCBL3
ThCBL3
CcCBL2
CsCBL2
PtCBL2-2
TcCBL2
GmCBL3
PvulCBL3-1
CsatCBL3
PtCBL2-1
RcCBL2
MgCBL3
MtCBL2-1
EgCBL2
GmCBL2-1
GmCBL2-2
PvulCBL3-2
PerCBL3
VvCBL3-2
GrCBL3-3
GrCBL3-2
TcCBL3
GrCBL3-1
MtCBL3-1
BdCBL3
OsCBL3-3
SiCBL3-3
ZmCBL3-1
OsCBL3-1
PvCBL3-1
PhCBL2
SiCBL3-1
CcCBL3
CsCBL3
MgCBL3-2
SlCBL3-2
StCBL3-4
CsatCBL2
MeCBL3
RcCBL3
PtCBL2-3
PtCBL3
StCBL3-1
SlCBL3-1
StCBL3-2
MdCBL1-2
MdCBL2

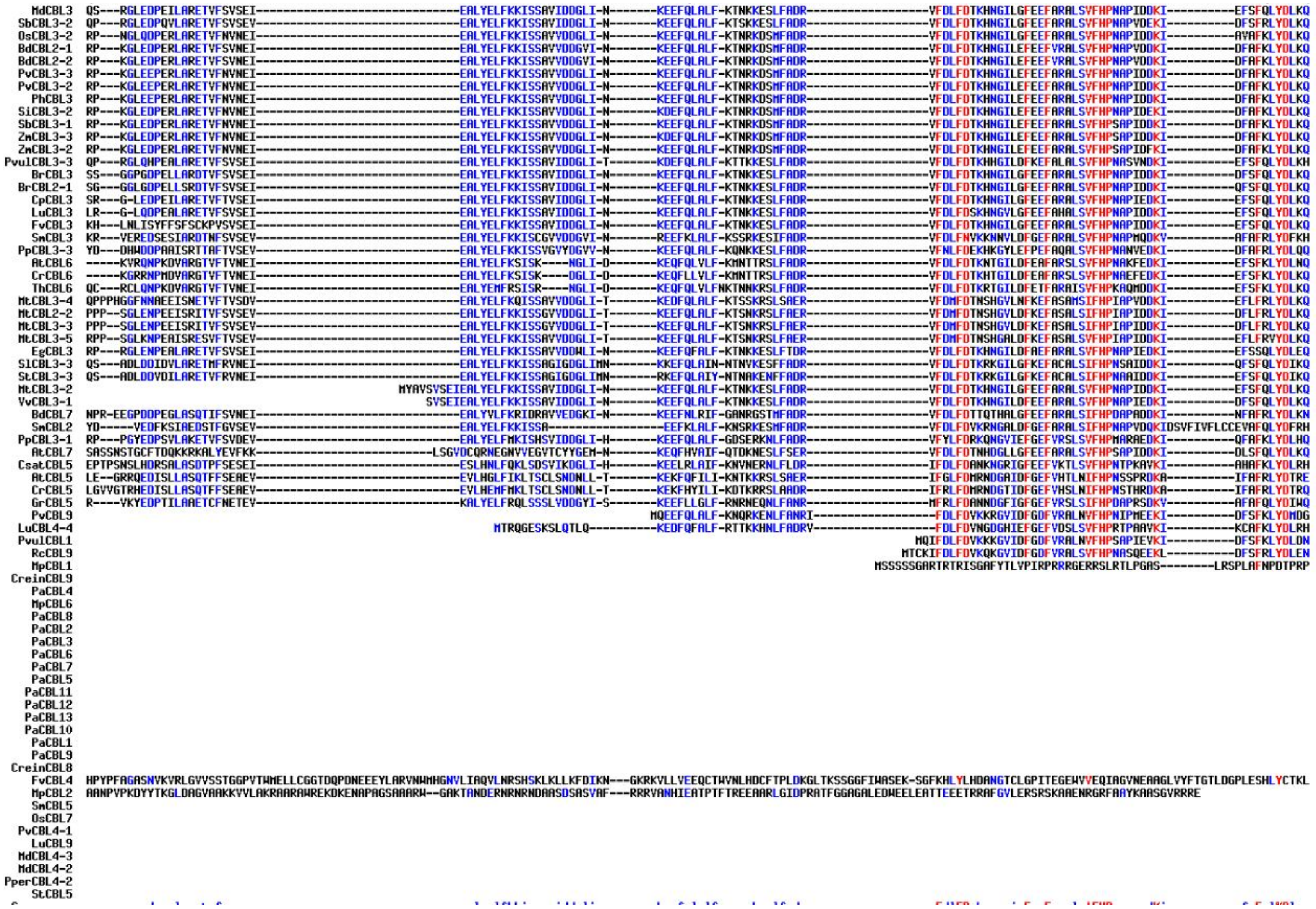
MdCBL3
SbCBL3-2
OsCBL3-2
BdCBL2-1
BdCBL2-2
PvCBL3-3
PvCBL3-2
PhCBL3
SiCBL3-2
SbCBL3-1
ZmCBL3-3
ZmCBL3-2
PvulCBL3-3
BrCBL3
BrCBL2-1
CpCBL3
LuCBL3
FvCBL3
SmCBL3
PpCBL3-3
AtCBL6
CrCBL6
ThCBL6
MtCBL3-4
MtCBL2-2
MtCBL3-3
MtCBL3-5
EgCBL3
SlCBL3-3
StCBL3-3
MtCBL3-2
VvCBL3-1
BdCBL7
SmCBL2
PpCBL3-1
AtCBL7
CsatCBL5
AtCBL5
CrCBL5
GrCBL5
PvCBL9
LuCBL4-4
PvulCBL1
RcCBL9
MpCBL1
CreinCBL9
PaCBL4
MpCBL6
PaCBL8
PaCBL2
PaCBL3
PaCBL6
PaCBL7
PaCBL5
PaCBL11
PaCBL12
PaCBL13
PaCBL10
PaCBL1
PaCBL9
CreinCBL8
FvCBL4
MpCBL2
SmCBL5
OsCBL7
PvCBL4-1
LuCBL9
MdCBL4-3
MdCBL4-2
PperCBL4-2
StCBL5

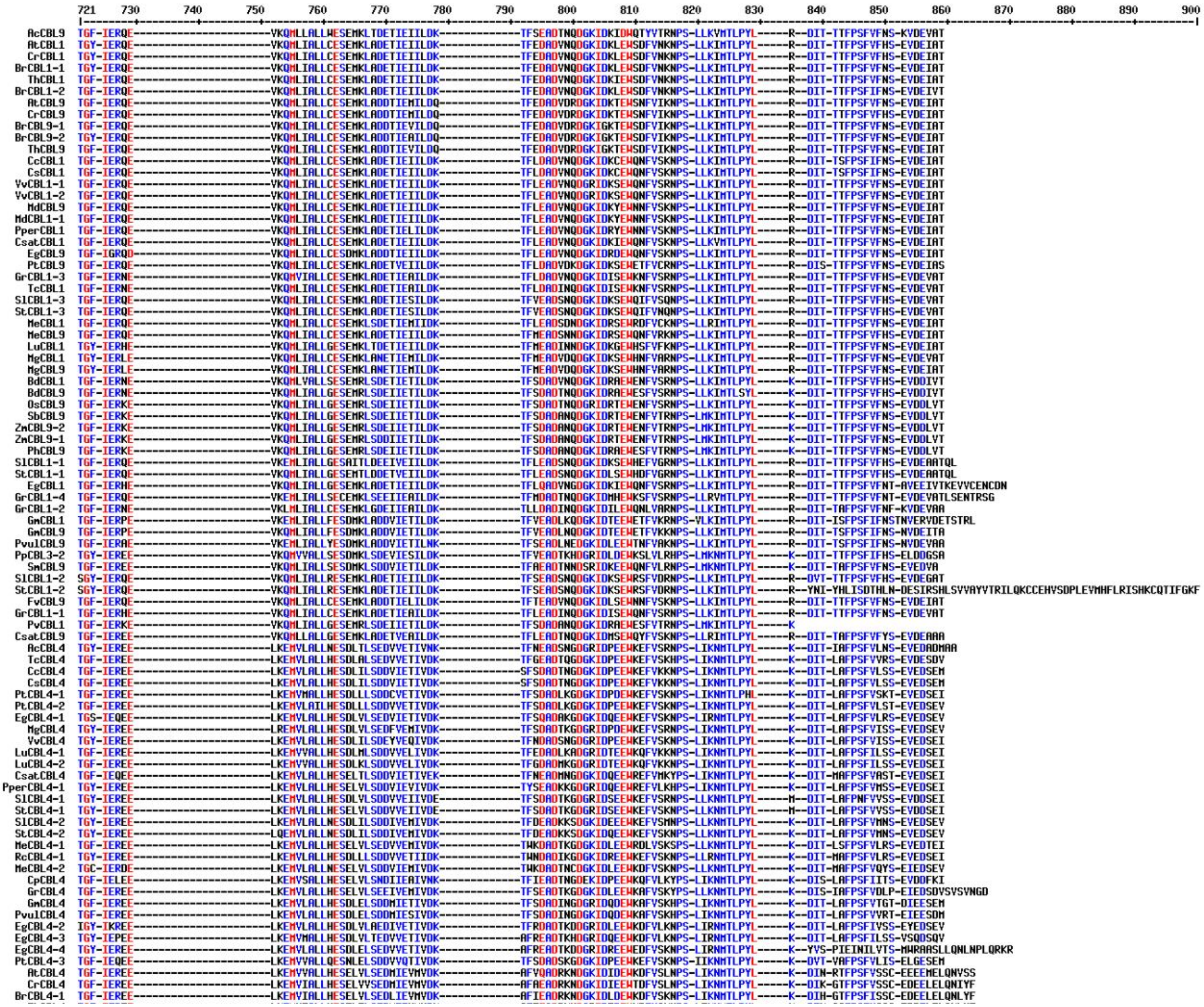

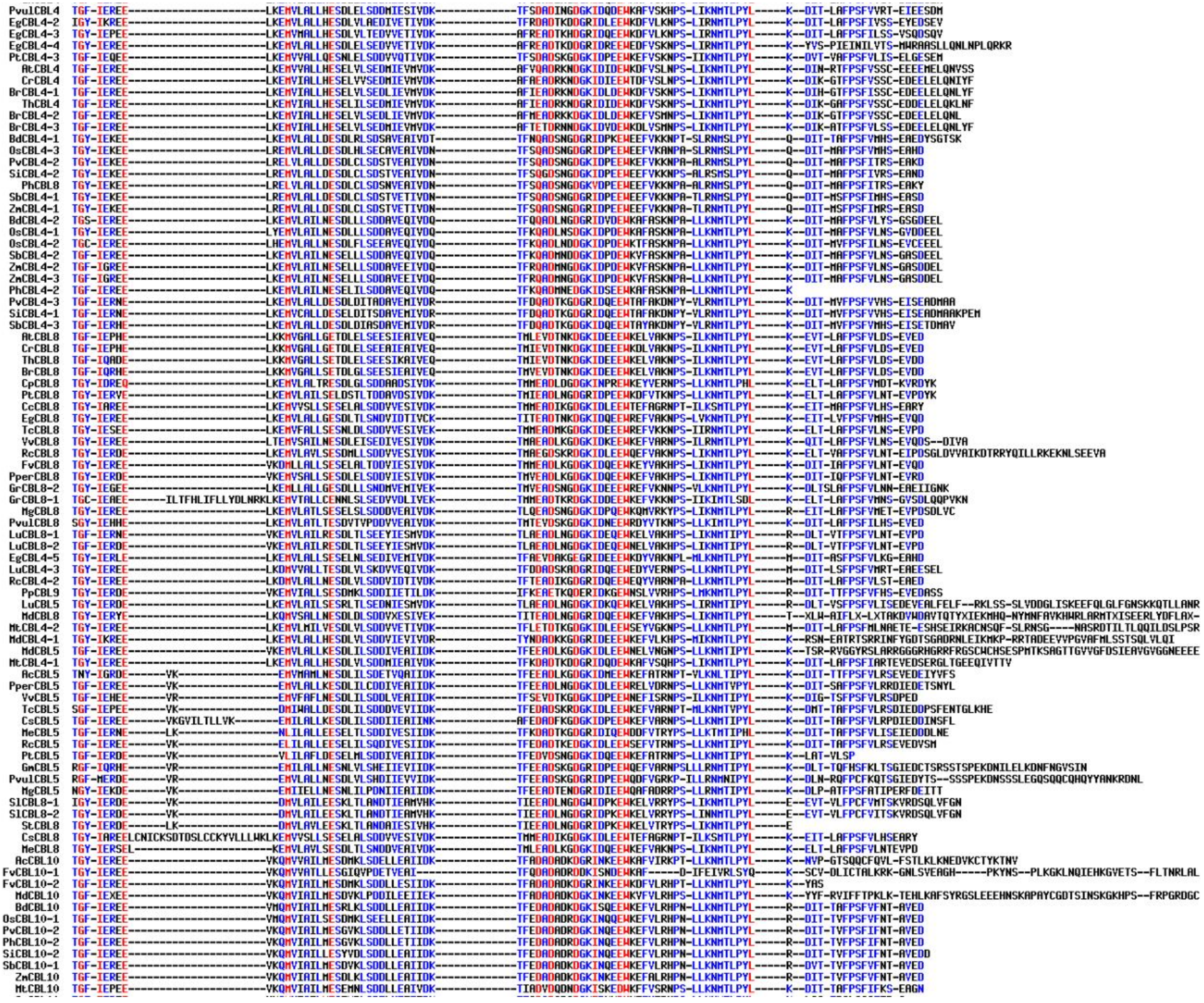
PvulCBL4
EgCBL4-2
EgCBL4-3
EgCBL4-4
PtCBL4-3
AtCBL4
CrCBL4
BrCBL4-1
ThCBL4
BrCBL4-2
BrCBL4-3
BdCBL4-1
OsCBL4-3
PvCBL4-2
SiCBL4-2
PhCBL8
SbCBL4-1
ZmCBL4-1
BdCBL4-2
OsCBL4-1
OsCBL4-2
SbCBL4-2
ZmCBL4-2
ZmCBL4-3
PhCBL4-2
PvCBL4-3
SiCBL4-1
SbCBL4-3
AtCBL8
CrCBL8
ThCBL8
BrCBL8
CpCBL8
PtCBL8
CcCBL8
EgCBL8
TcCBL8
VvCBL8
RcCBL8
FvCBL8
PperCBL8
GrCBL8-2
GrCBL8-1
MgCBL8
PvulCBL8
LuCBL8-1
LuCBL8-2
EgCBL4-5
LuCBL4-3
RcCBL4-2
PpCBL9
LuCBL5
MdCBL8
MtCBL4-2
MdCBL4-1
MdCBL5
MtCBL4-1
AcCBL5
PperCBL5
VvCBL5
TcCBL5
CsCBL5
MeCBL5
RcCBL5
PtCBL5
GmCBL5
PvulCBL5
MgCBL5
SlCBL8-1
SlCBL8-2
StCBL8
CsCBL8
MeCBL8
AcCBL10
FvCBL10-1
FvCBL10-2
MdCBL10
BdCBL10
OsCBL10-1
PvCBL10-2
PhCBL10-2
SiCBL10-2
SbCBL10-1
ZmCBL10
MtCBL10

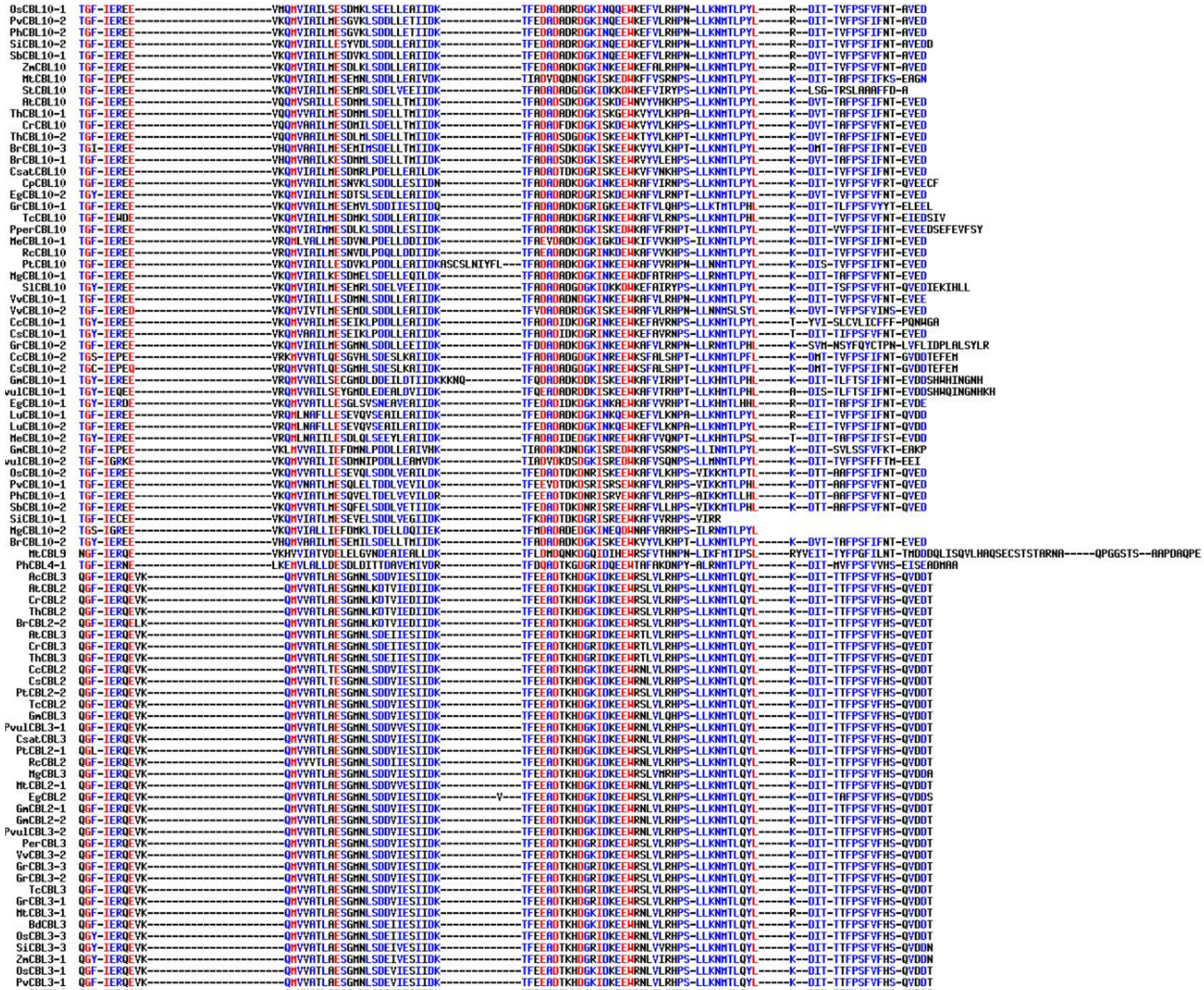

```
[illegible]
SiCBL3-1   QGF-IERQEVK----------------------QMVVATLAESGMNLSDEVIESIIDK-------------TFEEADTKHDGKIDKEEWRNLVLRHPS-LLKNMTLQYL-----K--DIT-TTFPSFVFHS-QVDDT
CcCBL3     QGF-IERQEVK----------------------QMVVATLAESGMNLSDDVIETIIDK-------------TFEEADTKHDGKIDKEEWRSLVLRHPS-LLKNMTLQYL-----K--DIT-TTFPSFVFHS-RVEDT
CsCBL3     QGFFIERQEVK----------------------QMVVATLAESGMNLSDDVIETIIDK-------------TFEEADTKHDGKIDKEEWRSLVLRHPS-LLKNMTLQYL-----K--DIT-TTFPSFVFHS-RVEDT
MgCBL3-2   QGF-IERQEVK----------------------QMVVATLAESGMNLSDEVIESIIDK-------------TFEEADTKHDGKIDKEEWRSLVLRHPS-LLKNMTLQYL-----K--DIT-TTFPSFVFHS-RVEDT
SlCBL3-2   QGF-IERQEVK----------------------QMVVATLAESGMNLSDDVIESIIDK-------------TFEEADTKHDGKIDKEEWRNLVLQHPS-LLKNMTLQYL-----K--DIT-TTFPSFVFHS-RVEDT
StCBL3-4   QGF-IERQEVK----------------------QMVVATLAESGMNLSDDVIESIIDK-------------TFEEADTKHDGKIDKEEWRNLVLQHPS-LLKNMTLQYL-----K--DIT-TTFPSFVFHS-RVEDT
CsatCBL2   QGF-IERQEVK----------------------QMVVATLAESGMNLSDDVIESIIDK-------------TFEEADTKHDGKIDKEEWRNLVMRHPS-LLKNMTLQYL-----K--DIT-TTFPSFVFHS-QVDDT
MeCBL3     QGF-IERQEVK----------------------QMVVATLAESGMNLSDDVIESIIDK-------------TFEEADTKHDGKIDKEEWRNLVLRHPS-LLKNMTLQYL-----K--DIT-TTFPSFVFHS-QVDDM
RcCBL3     QGF-IERQEVK----------------------QMVVATLAESGMNLSDDVIESIIDK-------------TFEEADTKHDGKIDKEEWRSLVLRHPS-LLKNMTLQYL-----K--DIT-TTFPSFVFHS-QVDDT
PtCBL2-3   QGF-IERQEVK----------------------QMVVATLAESGMNLSDDVIESIIDK-------------TFEEADTKHDGKIDKEEWRSLVLRHPS-LLKNMTLQYL-----K--DIT-TTFPSFVFHS-QVDDT
PtCBL3     QGF-IERQEVK----------------------QMVVATLAESGMNLSDDVIESIIDK-------------TFEEADTKHDGKIDKEEWRSLVLRHPS-LLKNMTLQYL-----K--DIT-TTFPSFVFHS-QVDDT
StCBL3-1   QGF-IERQEVK----------------------QMVVATLAETGMNLSDEIIESIIDK-------------TFEEADTKHDGKIDKEEWRNLVMRHPS-LLKNMTLQYL-----K--DIT-TTFPSFVFHS-RVPDT
SlCBL3-1   QGF-IERQEVK----------------------QMVVATLAESGMNLSDDVIESIIDK-------------TFEEADTKHDGKIDKEEWRNLVLRHPS-LLKNMTLQYL-----K--DIT-TTFPSFVFHS-RVEDT
StCBL3-2   QGF-IERQEVK----------------------QMVVATLAESGMNLSDDVIESIIDK-------------TFEEADTKHDGKIDKEEWRNLVLRHPS-LLKNMTLQYL-----K--DIT-TTFPSFVFHS-RVEDT
MdCBL1-2   TGF-IERQEVK----------------------QMLIALLCESEMKLADETIEIILDK-------------TFLEADVNQDGKIDKYEWNKFVSKNPS-LLKIMTLPYL-----R--DIT-TTFPSFVFNS-EVDEIAT
MdCBL2     QGF-IERQEVK----------------------QMVVATLAESGMNLSDDVIESIIDK-------------TFEEADTKHDGRIDKEEWRSLVLRHPS-LLKNMTLQYL-----K--DIT-TTFPSFVFHS-QVDDT
MdCBL3     QGF-IERQEVK----------------------QMVVATLAESGMNLSDDVIESIIDK-------------TFEEADTKHDGRIDKEEWRSLVLRHPS-LLKNMTLQYL-----K--DIT-TTFPSFVFHS-QVDDT
SbCBL3-2   QGY-IERQEVK----------------------QMVVATLAESGMNLSDEIVESIIDK-------------TFEEADTKHDGRIDKEEWRSLVIRHPS-LLKNMTLQYL-----K--DIT-TTFPSFVFHS-QVDDN
OsCBL3-2   QGF-IEKQEVK----------------------QMVVATLAESGMNLSDEIIEGIIDK-------------TFEEADTKHDGKIDKEEWRNLVLRHPS-LLKNMTLPYL-----R--DIT-TTFPSFVFNS-QVEDA
BdCBL2-1   QGF-IEKQEVK----------------------QMVVATLAESGMNLSDEVIEGIIDK-------------TFEEADTKHDGKIDREEWRNLVLRHPS-LLKNMTLPYL-----R--DIT-TTFPSFVFNS-QVEDA
BdCBL2-2   QGF-IEKQEVK----------------------QMVVATLAESGMNLSDEVIEGIIDK-------------TFEEADTKHDGKIDKEEWRNLVLRHPS-LLKNMTLPYL-----R--DIT-TTFPSFVFNS-QVEDA
PvCBL3-3   QGF-IEKQEVK----------------------QMVVATLAESGMNLSDDIIEGIIDK-------------TFEEADTKHDGKIDKEEWRNLVLRHPS-LLKNMTLPYL-----R--DIT-TTFPSFVFNS-QVEDA
PvCBL3-2   QGF-IEKQEVK----------------------QMVVATLAESGMNLSDDIIEGIIDK-------------TFEEADTKHDGKIDKEEWRNLVLRHPS-LLKNMTLPYL-----R--DIT-TTFPSFVFNS-QVEDA
PhCBL3     QGF-IEKQEVK----------------------QMVVATLAESGMNLSDDIIEGIIDK-------------TFEEADTKHDGKIDKEEWRNLVLRHPS-LLKNMTLPYL-----R--DIT-TTFPSFVFNS-QVEDA
SiCBL3-2   QGF-IEKQEVK----------------------QMVVATLAESGMNLSDDIIEGIIDK-------------TFEEADTKHDGKIDKEEWRNLVLRHPS-LLKNMTLPYL-----R--DIT-TTFPSFVFNS-QVEDA
SbCBL3-1   QGF-IEKQEVK----------------------QMVVATLAESGMNLSDDIIEGIIDK-------------TFEEADTKHDGKIDKEEWRSLVLRHPS-LLKNMTLPYL-----R--DIT-TTFPSFVFNS-QVEDA
ZmCBL3-3   QGF-IEKQEVK----------------------QMVVATLAESGMNLSDDIIEGIIDK-------------TFEEADTKHDGKIDKEEWRSLVLRHPS-LLKNMTLPYL-----R--DIT-TTFPSFVFNS-QVEDA
ZmCBL3-2   QGF-IEKQEVK----------------------QMVVATLAESGMNLSDDIIEGIIDK-------------TFEEADTKHDGKIDKEEWRSLVLRHPS-LLKNMTLPYL-----R--DIT-TTFPSFVFNS-QVEDA
PvulCBL3-3 QGF-IQRQELK----------------------QMVVATLSEAGMNLADDVIESIIDK-------------TFEEADTKHDGKIDKEEWRNLVLRHPS-LLRNMTLQYL-----K--DIT-TTFPSFVFHS-QVDDV
BrCBL3     QGF-IERQEVK----------------------QMVVATLAESGMNLSDE---------------------TFEEADTKHDGRIDKEEWRILVLRHPS-LLKNMTLQYL-----K--DIT-TTFPSFVFHS-QVEDT
BrCBL2-1   QGF-IERQEVK----------------------QMVVATLAESGMNLKDTVIEDIIDK-------------TFEEADTKHDGKIDKEEWRSLVLRHPS-LLKNMTLQYL-----K
CpCBL3     QGF-IERQEVK----------------------QMVVATLAESGMNLSDDVIESIIDK-------------TFEEADTKHDGRIDKEEWRSLVLRHPS-LLKNMTLQYL-----K
LuCBL3     QGF-IERQEVK----------------------QMVVATLAESGMNLSDDVIESIIDK-------------TFEEADTKHDGKIDKEEWRSLVLRHPS-LLKNMTLQYL-----K
FvCBL3     QGF-IERQEVK----------------------QMVVATLAESGMNLSDEVIESIIDK-------------TFEEADTKHDGRIDKEEWRSLVLRHPS-LLKNMTLQYL-----K--DIT-TTFPSFVFHS-QVDDT
SmCBL3     QGY-IDRGEVK----------------------RMVVATLAESGMNLSDEVVENIIDK-------------TFEEADTKRDGMIDEEEWSNLVQKHPS-LLKNMSLLYL-----K--DIT-TTFPSFVFHS-QVEDT
PpCBL3-3   QGY-IERSEVK----------------------RMVVATLAESGLNLSDDVIEEIIDK-------------TFLEADTKNDGRIDKEEWRTLVMQHPS-LLKNMTLPYL-----K--DIT-TTFPSFIFHS-LVEET
AtCBL6     QGY-IKRQEVK----------------------QMVVRTLAESGMNLSDHVIESIIDK-------------TFEEADTKLDGKIDKEEWRSLVLRHPS-LLQNMSLQHL-----K--DVT-KTFPNFVFHT-IVTDTPSELDG
CrCBL6     QGS-IERHEVK----------------------QMVVATLAESGMNLSDHVIESIIDK-------------TFEEADTKLDGKIDKEEWRSLVLRHPS-LLQNMSLQHL-----K--DVT-KTFPNFVFHT-IVSDTPSELDR
ThCBL6     QGY-IERDELK----------------------QMVVAILAESGMNLSDDVIESIIDK-------------TFEEADSKLDGKIDKEEWQSLVLLHPS-LLQNMTLQHL-----K--DIT-KTFTHFVFHT-IVSDNPSEI
MtCBL3-4   QGY-IDREQLK----------------------EMVVATLSESGVQLSDYVINRIIDK-------------TFEDADRNHDGKIDKEEFYNLALRHPS-LLKNMSLLYL-----T--EIT-TKFPSFVFHS-QVEDTEASALSC
MtCBL2-2   QGY-IDRERLK----------------------QMVVATLSEFGMRLTEEMLNSIMDK-------------TFEEVDTNHDGKIDREEWKNLAMQQTS-LLKNMTLPYL-----T--EIT-TTFPSFIFHT-QVDDD
MtCBL3-3   QGY-IDREQLK----------------------QMVVATLSESGMRLTEEMLNSIMDK-------------TFEEVDTNHDGKIDREEWKNLAMQQTS-LLKNMTLPYL-----T--EIT-TTFPSFIFHT-QVDDD
MtCBL3-5   QGY-IERDELK----------------------QLVVATLSESDMELSDEMINGIIDK-------------TFEEVDTNHDGKIDKEEWKNLAMQQPS-LLTNMTLPYL-----T--EIT-TAFPSFIFHT-QVDDN
EgCBL3     QGF-IERQEVK----------------------QMVVATLAESGMNLSDDVIESIIDK-------------TFEEADTKHNGKIDNEEWRRLVLRHPS-LFYKT
SlCBL3-3   QGF-IEREEVK----------------------QMMLVALAQSNMKFSDNVIERIIDN-------------TFEEADINRDGKIDKEEWRSLVLRNPS-LLKNMTLPFL-----T--DIT-TMFPNFTYHS-RVED
StCBL3-3   QGF-IERQEVK----------------------QMMLVALAETNMKLSDNVIESIIDH-------------TFEEADTNRDGKIDKEEWRSLVLRNPS-LLQNMTLPYL-----T--DIT-TMFPSFVFHS-RVEDDLIWD
MtCBL3-2   QGF-IERQEVK----------------------QMVVATLAESGMNLSDDVIESIIDK-------------TFEEADTKHDGKIDKEEWRNLVLRHPS-LLKNMTLQYL-----K--DIT-TTFPSFVFHS-QVDDT
VvCBL3-1   QGF-IERQEVK----------------------QMVVATLAESGMNLSDDVIESIIDK-------------TFEEADTKHDGKIDKEEWRSLVLRHPS-LLKNMTLQYL-----K--DIT-TTFPSFVFHS-QVDDT
BdCBL7     QGF-IQKQELK----------------------QMMEATLAESNLDLSDEVIDTIIDK-------------TFEEADTKKDGKIDIDEWRNLVNVHPS-LLKNMTLTYL-----R--DVT-MTFPEFVFHS-RLRET
SmCBL2     EGF-IQRSEVK----------------------RMVVATLAESGVNLSDEVVENIIDK-------------TFEEADTKHDGKIDKDEWRSLVQHHPS-LLKNMNLPYL-----K--DIT-TTFPSFIFYS-QVEDT
PpCBL3-1   TGY-IEREEVK----------------------EMLDAILCESTLDLSPEVIETILDM-------------TFAEAGTKLAGRIDEEEWERLVRRHPG-LMQNMTLPYL-----R--DII-TAFPSFSRKI-DKD
AtCBL7     QGF-IERQGVK----------------------QLVVATLAASGMSQSDEIVESIIDK-------------TFVQADTKHEGMIDEEEWMDLVFRHPL-LLKNMTLQYL-----K--DIT-TTFPSFVLHS-QVEDT
CsatCBL5   TGF-IEREELR----------------------EMVLALLCESDLVLPDDVVKMIVDK-------------TFNEADAKGDGKIDEEEWKAYVAQNPS-LLKHMTLPYL-----M--YVS-NLSSKSYKYS-QILEN
AtCBL5     TGF-IEPEEVK----------------------EMIIDVLEESELMLSESIIDSIVSK-------------TFEEADWKKDGIIDLEEWENFVATYPL-TLKNMTIPFL-----K--DIP-RIFPTFLR
CrCBL5     TGF-IEPEVVK----------------------EMIIDVLEESELVLSESIIDSIVSK-------------TFEEADWKKDGKIDLEEWENFVATYPL-TLKNMTIPFL-----K--DIP-RIFPTLFW
GrCBL5     TGF-IEPEEVKGILWTSLIGYYHDPLTSEVK--EMILALLNESNLVLSNDVIDVIVDK-------------TFKDADSNKDGKIDMKEWDEFVARNPI-LMKNMTIPHL-----M--DLT
PvCBL9     TGF-IERKE----------------------VKQMLIALLGESEMRLSDDIIETILDK-------------TFSDADANQDGKIDRAEWESFVTRNPS-LMKIMTLPYL-----K--DIT-TTFPSFVFNS-EVDDLVT
LuCBL4-4   TGY-IEREE----------------------LKDMVVALLTESDLVLSEDVVEQIVDKVLNFEPLKIALPATCDDADSKGDGRIDQEEWEDYVEKNPS-LLKNMTLPYLMLTLKNQKDIT-LSFPSFVMRT-EAEESEL
PvulCBL1   TGF-IERHE----------------------VKQMINALLCEAEIKLSDDMIETIINK-------------TFMDADPNQDGKIDKYEWKNFVFENPS-LLKVMTLPYL-------RDVT-TAFPSFVFHS-KVEDEITE
RcCBL9     TGF-IERQE----------------------VKQMLIALLCESEMKLSDETIEIILDK-------------TFLEADADSDGKIDRSEWKNFVCKNPS-LLKIMTLPYL-------RDIT-TTFPSFVFNS-EVDEIAT
MpCBL1     LSTPTDAYELHPDIALY-GTTLSVD------AREFAQFFDIERSNVFLPRLVALFDVSGDGHMSLYEFVVCLAQFSVNKKQSEHVYFAWRLFDTDDSGAMTKDEFVEALSGAFILITLVPIRPRWRCELHSLRTFSPGVPSYARAGTAKVITSDRRGRGLANFGNVGGVAKGIDTIMREM
CreinCBL9
PaCBL4
MpCBL6
PaCBL8
PaCBL2
PaCBL3
PaCBL6
PaCBL7
PaCBL5
[illegible]
```
