# Supplementary material for: Genome-wide identification of Calcineurin B-Like (CBL) gene family of plants reveals novel conserved motifs and evolutionary aspects in calcium signaling events
Source: BMC Plant Biol. 2015 Aug 6;15:189. doi: 10.1186/s12870-015-0543-0 (PMC4527274; doi:10.1186/s12870-015-0543-0)
Supplement: Additional file 2: — Table representing molecular mass (kDa) and isoelectric point of different CBL genes from 38 plant species identified during this study. [file 12870_2015_543_MOESM2_ESM.pdf]

Additional Data File 2

Table representing molecular mass (in kDa) and isoelectric point of different CBL genes from 41 plant species identified during this study

Gene Name	Locus ID	Mol. Weight (kD)	pI
AcCBL3	Aquca_002_00627	29.620	5.14
AcCBL4	Aquca_037_00212	24.475	4.83
AcCBL5	Aquca_037_00167	24.559	4.70
AcCBL9	Aquca_053_00098	24.455	4.86
AcCBL10	Aquca_015_00409	33.001	5.58
Arabidopsis thaliana			
AtCBL1	AT4G17615	24.553	4.48
AtCBL2	AT5G55990	25.809	4.65
AtCBL3	AT4G26570	26.537	4.55
AtCBL4	AT5G24270	25.693	4.87
AtCBL5	AT4G01420	23.517	4.56
AtCBL6	AT4G16350	26.028	5.65
AtCBL7	AT4G26560	24.404	4.53
AtCBL8	AT1G64480	24.649	4.76
AtCBL9	AT5G47100	24.531	4.32
AtCBL10	AT4G33000	29.342	4.46
Brachypodium distachyon			
BdCBL1	Bradi1g78640	24.412	4.83
BdCBL2-1	Bradi4g41910	25.742	4.96
BdCBL2-2	Bradi1g28160	25.728	4.96
BdCBL3	Bradi4g02740	25.767	4.98
BdCBL4-1	Bradi2g18740	24.704	5.10
BdCBL4-2	Bradi3g10640	24.358	4.95
BdCBL7	Bradi3g43510	25.485	4.82
BdCBL9	Bradi3g33700	24.446	4.89
BdCBL10	Bradi2g41730	36.409	5.91
Brassica rapa			
BrCBL1-1	Bra040169	24.586	4.82
BrCBL1-2	Bra012655	24.603	4.77

BrCBL2-1	Bra028949	25.151	5.11
BrCBL2-2	Bra035598	25.865	5.04
BrCBL3	Bra026421	24.885	4.96
BrCBL4-1	Bra009743	25.582	5.14
BrCBL4-2	Bra026462	25.482	5.00
BrCBL4-3	Bra029396	25.369	4.87
BrCBL8	Bra027703	24.751	5.37
BrCBL9-1	Bra022104	24.350	4.75
BrCBL9-2	Bra017504	24.350	4.75
BrCBL10-1	Bra034543	28.462	4.95
BrCBL10-2	Bra011404	24.335	4.77
BrCBL10-3	Bra037030	29.874	4.70
Capsella rubella			
CrCBL1	Carubv10005722m.g	24.481	4.87
CrCBL2	Carubv10027067m.g	25.843	5.04
CrCBL3	Carubv10005674m.g	26.031	4.93
CrCBL4	Carubv10001908m.g	25.653	5.14
CrCBL5	Carubv10003821m.g	23.881	5.24
CrCBL6	Carubv10005675m.g	26.007	5.50
CrCBL8	Carubv10022024m.g	24.678	5.17
CrCBL9	Carubv10027124m.g	24.496	4.80
CrCBL10	Carubv10006225m.g	27.689	4.79
Carica papaya			
CpCBL3	evm.TU.supercontig_12.107	24.071	5.03
CpCBL4	evm.TU.supercontig_3.83	24.358	4.93
CpCBL8	evm.TU.supercontig_571.2	24.858	5.18
CpCBL10	evm.TU.supercontig_7.168	28.907	5.16
Chlamydomonas reinhardtii			
CreinCBL8	Cre08.g363750	14.291	4.23
CreinCBL9	Cre16.g650750	27.418	4.67
Citrus clementina			
CcCBL1	Ciclev10022219m.g	24.435	4.81
CcCBL2	Ciclev10016575m.g	26.043	4.84
CcCBL3	Ciclev10009412m.g	25.668	4.93
CcCBL4	Ciclev10022148m.g	25.110	4.87

CcCBL8	Ciclev10023612m.g	24.442	5.01
CcCBL10-1	Ciclev10009236m.g	29.685	4.85
CcCBL10-2	Ciclev10026324m.g	28.954	4.66
Citrus sinensis			
CsCBL1	orange1.1g040252m.g	24.421	4.81
CsCBL2	orange1.1g027239m.g	26.057	4.84
CsCBL3	orange1.1g027336m.g	25.815	4.93
CsCBL4	orange1.1g027657m.g	25.110	4.87
CsCBL5	orange1.1g041619m.g	25.360	5.11
CsCBL8	orange1.1g048069m.g	24.315	4.87
CsCBL10-1	orange1.1g025162m.g	29.410	4.71
CsCBL10-2	orange1.1g025241m.g	29.000	4.71
Cucumis sativus			
CsatCBL1	Cucsa.321410	24.533	4.69
CsatCBL2	Cucsa.044480	25.655	4.87
CsatCBL3	Cucsa.041730	25.882	4.88
CsatCBL4	Cucsa.032100	24.530	4.84
CsatCBL5	Cucsa.259240	24.667	5.64
CsatCBL9	Cucsa.313710	21.460	4.72
CsatCBL10	Cucsa.395140	28.318	4.85
Eucalyptus grandis			
EgCBL1	Eucgr.D02136	25.387	5.00
EgCBL2	Eucgr.F03674	26.062	4.83
EgCBL3	Eucgr.E03829	23.003	4.95
EgCBL4-1	Eucgr.A01457	24.323	4.86
EgCBL4-2	Eucgr.K00375	24.483	4.92
EgCBL4-3	Eucgr.K00377	24.255	4.96
EgCBL4-4	Eucgr.K00380	25.761	5.28
EgCBL4-5	Eucgr.E00051	24.656	4.91
EgCBL8	Eucgr.D01757	24.655	4.89
EgCBL9	Eucgr.E00335	24.403	4.92
EgCBL10-1	Eucgr.F03125	28.032	5.51
EgCBL10-2	Eucgr.C00642	29.032	4.85
Fragaria vesca			
FvCBL3	gene01496-v1.0-hybrid	23.389	5.14

FvCBL4	gene12873-v1.0-hybrid	115.266	5.97
FvCBL8	gene32137-v1.0-hybrid	24.980	5.01
FvCBL9	gene10766-v1.0-hybrid	31.855	6.81
FvCBL10-1	gene23084-v1.0-hybrid	42.436	5.85
FvCBL10-2	gene02679-v1.0-hybrid	35.693	5.20
Glycine max			
GmCBL1	Glyma17g15893	28.256	5.12
GmCBL2-1	Glyma08g44580	26.037	4.83
GmCBL2-2	Glyma18g08230	26.065	4.88
GmCBL3	Glyma07g39936	25.777	4.78
GmCBL4	Glyma06g13420	25.543	4.79
GmCBL5	Glyma08g02740	26.725	4.90
GmCBL9	Glyma05g05580	24.416	4.87
GmCBL10-1	Glyma08g20700	30.778	5.14
GmCBL10-2	Glyma17g34761	29.255	4.84
Gossipium raimondii			
GrCBL1-1	Gorai.003G178700	24.450	4.84
GrCBL1-2	Gorai.004G191400	23.862	4.81
GrCBL1-3	Gorai.007G030300	24.375	4.86
GrCBL1-4	Gorai.006G214700	25.392	5.10
GrCBL3-1	Gorai.013G150400	25.961	4.94
GrCBL3-2	Gorai.009G450400	25.982	4.93
GrCBL3-3	Gorai.002G102900	25.882	4.98
GrCBL4	Gorai.007G015400	24.914	4.87
GrCBL5	Gorai.008G255800	24.581	4.80
GrCBL8-1	Gorai.008G255900	27.260	5.29
GrCBL8-2	Gorai.006G207100	24.482	5.15
GrCBL10-1	Gorai.010G101400	29.255	4.86
GrCBL10-2	Gorai.009G045600	30.312	5.07
Linum usitatissimum			
LuCBL1	Lus10011028.g	24.425	5.01
LuCBL3	Lus10038764.g	23.771	5.02
LuCBL4-1	Lus10018108.g	24.629	4.87
LuCBL4-2	Lus10022407.g	24.532	4.92
LuCBL4-3	Lus10030252.g	24.795	4.93

LuCBL4-4	Lus10004006.g	22.316	4.83
LuCBL5	Lus10023069.g	45.258	5.27
LuCBL8-1	Lus10001816.g	24.732	5.48
LuCBL8-2	Lus10003191.g	24.891	5.17
LuCBL9	Lus10032400.g	20.348	5.64
LuCBL10-1	Lus10015630.g	28.316	4.78
LuCBL10-2	Lus10037648.g	28.275	4.83
Malus domestica			
MdCBL1-1	MDP0000207134	24.416	4.87
MdCBL1-2	MDP0000262916	79.456	9.40
MdCBL2	MDP0000119547	47.470	7.94
MdCBL3	MDP0000137075	47.470	7.94
MdCBL4-1	MDP0000155124	32.594	5.77
MdCBL4-2	MDP0000774066	16.236	4.62
MdCBL4-3	MDP0000263313	16.385	4.82
MdCBL5	MDP0000294682	41.463	7.77
MdCBL8	MDP0000256696	39.019	6.24
MdCBL9	MDP0000259502	24.416	4.87
MdCBL10	MDP0000186655	49.290	8.30
Manihut esculenta			
MeCBL1	cassava4.1_016071m.g	24.559	4.75
MeCBL3	cassava4.1_023888m.g	25.836	4.88
MeCBL4-1	cassava4.1_015878m.g	25.360	5.31
MeCBL4-2	cassava4.1_022392m.g	25.697	4.71
MeCBL5	cassava4.1_029089m.g	24.326	4.64
MeCBL8	cassava4.1_023193m.g	20.977	4.72
MeCBL9	cassava4.1_016083m.g	24.497	4.87
MeCBL10-1	cassava4.1_014701m.g	28.741	4.82
MeCBL10-2	cassava4.1_014733m.g	28.559	4.83
Medicago truncatula			
MtCBL2-2	AC233669_22	25.972	4.90
MtCBL2-3	Medtr2g027480	26.307	4.95
MtCBL3-1	Medtr5g096420	26.064	4.83
MtCBL3-2	Medtr3g060730	21.695	4.90
MtCBL3-3	Medtr2g027440	26.171	4.79

MtCBL3-4	Medtr2g027520	27.082	4.88
MtCBL3-5	Medtr2g027500	25.682	4.79
MtCBL4-1	Medtr3g091440	27.814	5.29
MtCBL4-2	AC235758_37	35.072	5.24
MtCBL9	Medtr4g113510	46.431	9.61
MtCBL10	Medtr1g016430	33.099	4.91
Micromonas pusila			
MpCBL1	MicpuC2.EuGene.0000130349	25.972	4.90
MpCBL2	MicpuC2.EuGene.0000040369	70.365	9.51
MpCBL6	e_gw1.15.503.1	21.846	5.49
Mimulus guttatus			
MgCBL1	mgv1a024164m.g	24.458	4.80
MgCBL3-1	mgv1a013241m.g	25.969	4.83
MgCBL3-2	mgv1a013314m.g	25.800	4.94
MgCBL4	mgv1a013679m.g	24.647	4.87
MgCBL5	mgv1a023934m.g	24.434	4.86
MgCBL8	mgv1a013491m.g	24.916	4.64
MgCBL9	mgv1a022772m.g	23.474	4.74
MgCBL10-1	mgv1a012532m.g	28.329	4.85
MgCBL10-2	mgv1a019977m.g	28.718	5.13
Oryza sativa			
OsCBL3-1	LOC_Os03g42840	25.803	4.508
OsCBL3-2	LOC_Os12g06510	33.479	4.79
OsCBL3-3	LOC_Os12g40510	25.864	4.54
OsCBL4-1	LOC_Os02g18880	24.399	4.52
OsCBL4-2	LOC_Os02g18930	24.491	4.65
OsCBL4-3	LOC_Os05g45810	23.916	4.71
OsCBL7	LOC_Os02g27940	19.311	4.24
OsCBL9	LOC_Os10g41510	24.496	4.40
OsCBL10-1	LOC_Os01g39770	32.892	4.58
OsCBL10-2	LOC_Os01g51420	29.915	4.61
Panicum halii			
PhCBL2	Pahal.0007s0427	25.771	4.94
PhCBL3	Pahal.0128s0167	25.727	4.96
PhCBL4-1	Pahal.0071s0163	21.305	5.32

PhCBL4-2	Pahal.0012s0184	22.417	5.24
PhCBL8	Pahal.0417s0014	23.907	4.94
PhCBL9	Pahal.0019s0042	24.353	4.77
PhCBL10-1	Pahal.0306s0048	29.789	5.10
PhCBL10-2	Pahal.0026s0062	34.076	5.38
Panicum vigatum			
PvCBL1	Pavirv00026000m.g	22.180	5.05
PvCBL3-1	Pavirv00044173m.g	25.785	4.94
PvCBL3-2	Pavirv00040240m.g	25.727	4.96
PvCBL3-3	Pavirv00003030m.g	25.757	4.96
PvCBL4-1	Pavirv00047304m.g	15.510	6.70
PvCBL4-2	Pavirv00048053m.g	23.701	4.77
PvCBL4-3	Pavirv00017512m.g	24.856	4.98
PvCBL9	Pavirv00036730m.g	18.796	4.77
PvCBL10-1	Pavirv00069048m.g	29.858	4.85
PvCBL10-2	Pavirv00016899m.g	37.204	5.73
Phaseolus vulgaris			
PvulCBL1	Phvul.002G002300	16.549	4.83
PvulCBL3-1	Phvul.003G088500	25.846	4.88
PvulCBL3-2	Phvul.008G086100	26.071	4.83
PvulCBL3-3	Phvul.006G135900	26.096	5.25
PvulCBL4	Phvul.009G052700	25.666	4.84
PvulCBL5	Phvul.002G299300	27.641	5.01
PvulCBL8	Phvul.002G299400	24.534	5.11
PvulCBL9	Phvul.003G225900	24.472	4.94
PvulCBL10-1	Phvul.010G139700	30.157	5.00
PvulCBL10-2	Phvul.001G034600	30.123	4.92
Physcomitrella Patens			
PpCBL3-1	Phpat.005G037800	27.374	4.90
PpCBL3-2	Phpat.001G148800	24.538	5.18
PpCBL3-3	Phpat.013G011100	26.628	4.91
PpCBL9	Phpat.017G068000	24.527	5.17
Picea abies			
PaCBL1	MA_9965p0020	17.798	5.18
PaCBL2	MA_27386p0010	22.260	4.40

PaCBL3	MA_10431324p0020	20.018	4.33
PaCBL4	MA_105065p0010	23.282	6.41
PaCBL5	MA_10204459p0010	21.223	4.54
PaCBL6	MA_269415p0010	14.687	4.40
PaCBL7	MA_3268p0010	21.272	4.36
PaCBL8	MA_7799814p0010	19.455	4.23
PaCBL9	MA_9445p0010	16.084	4.37
PaCBL10	MA_18112p0010	12.774	4.02
PaCBL11	MA_10280648p0010	21.632	4.54
PaCBL12	MA_184231p0010	20.126	5.42
PaCBL13	MA_10288665p0010	20.988	5.32
Populous trichocarpa			
PtCBL2-1	Potri.006G002900	25.887	4.90
PtCBL2-2	Potri.016G003500	25.894	4.84
PtCBL2-3	Potri.001G371700	25.672	4.93
PtCBL3	Potri.011G094900	25.769	4.88
PtCBL4-1	Potri.015G013100	26.014	5.07
PtCBL4-2	Potri.012G015100	24.498	4.88
PtCBL4-3	Potri.015G013200	24.009	4.78
PtCBL5	Potri.003G141400	24.994	4.70
PtCBL8	Potri.001G090200	24.660	4.77
PtCBL9	Potri.001G150200	24.340	4.79
PtCBL10	Potri.006G230200	30.234	4.96
Prunus persica			
PperCBL1	ppa011375m.g	24.542	4.87
PperCBL3	ppa011040m.g	26.052	4.94
PperCBL4-1	ppa011404m.g	24.475	4.88
PperCBL4-2	ppa016322m.g	16.091	4.52
PperCBL5	ppa014557m.g	24.820	4.82
PperCBL8	ppa020531m.g	25.206	5.05
PperCBL10	ppa010315m.g	29.509	4.74
Ricinus communis			
RcCBL2	29836.t000025	25.731	4.81
RcCBL3	27893.t000011	25.735	4.83
RcCBL4-1	30024.t000035	25.466	5.00

RcCBL4-2	29742.t000053	24.125	4.81
RcCBL5	30190.t000089	24.252	4.59
RcCBL8	30190.t000090	27.787	4.93
RcCBL9	29822.t000180	16.537	4.63
RcCBL10	29794.t000028	28.760	4.87
Selaginella moellendorffii			
SmCBL2	409467	26.721	4.99
SmCBL3	272115	26.182	5.29
SmCBL5	81220	24.311	5.34
SmCBL9	131200	24.314	4.80
Setaria italica			
SiCBL3-1	Si037393m.g	25.833	4.93
SiCBL3-2	Si011013m.g	25.614	4.96
SiCBL3-3	Si023163m.g	25.884	4.99
SiCBL4-1	Si002829m.g	25.256	4.99
SiCBL4-2	Si023239m.g	23.703	4.93
SiCBL10-1	Si004544m.g	27.857	5.44
SiCBL10-2	Si002269m.g	36.581	5.58
Solanum lycopersicum			
SlCBL1-1	Solyc06g060980.1	24.557	4.79
SlCBL1-2	Solyc08g007160.2	24.645	4.79
SlCBL1-3	Solyc08g077770.2	24.295	5.00
SlCBL3-1	Solyc07g065820.2	25.179	4.87
SlCBL3-2	Solyc12g015870.1	25.682	4.88
SlCBL3-3	Solyc02g032310.1	25.396	4.94
SlCBL4-1	Solyc06g051970.2	24.591	4.64
SlCBL4-2	Solyc03g083320.2	24.571	4.64
SlCBL8-1	Solyc00g034810.1	26.767	6.02
SlCBL8-2	Solyc12g055920.1	26.663	6.23
SlCBL10	Solyc08g065330.2	29.877	4.77
Solanum tuberosum			
StCBL1-1	PGSC0003DMG400026580	24.472	4.71
StCBL1-2	PGSC0003DMG400020493	30.356	5.80
StCBL1-3	PGSC0003DMG400004554	24.322	5.00
StCBL3-1	PGSC0003DMG400014370	25.515	4.99

StCBL3-2	PGSC0003DMG400022267	25.223	4.87
StCBL3-3	PGSC0003DMG400038238	25.903	4.79
StCBL3-4	PGSC0003DMG400029807	25.682	4.88
StCBL4-1	PGSC0003DMG400019601	24.618	4.76
StCBL4-2	PGSC0003DMG401009127	24.628	4.64
StCBL5	PGSC0003DMG400005840	13.610	4.61
StCBL8	PGSC0003DMG400017886	22.649	5.48
StCBL10	PGSC0003DMG400029942	32.745	4.79
Sorghum bicolor			
SbCBL3-1	Sobic.008G046500	25.676	4.96
SbCBL3-2	Sobic.008G152800	41.244	7.94
SbCBL4-1	Sobic.009G210300	24.047	4.77
SbCBL4-2	Sobic.004G130600	24.386	4.83
SbCBL4-3	Sobic.003G208400	25.107	5.05
SbCBL9	Sobic.001G294300	24.485	4.89
SbCBL10-1	Sobic.003G196400	35.866	5.29
SbCBL10-2	Sobic.003G275000	32.289	5.47
Thelluginella halophila			
ThCBL1	Thhalv10026227m.g	24.570	4.82
ThCBL2	Thhalv10014594m.g	25.829	5.04
ThCBL3	Thhalv10026170m.g	25.907	4.87
ThCBL4	Thhalv10004891m.g	25.393	5.22
ThCBL6	Thhalv10027149m.g	26.378	5.27
ThCBL8	Thhalv10023688m.g	24.636	5.27
ThCBL9	Thhalv10001023m.g	24.384	4.75
ThCBL10-1	Thhalv10026019m.g	30.015	5.16
ThCBL10-2	Thhalv10028908m.g	28.948	4.81
Theobroma cacao			
TcCBL1	Thecc1EG015835	24.298	4.86
TcCBL2	Thecc1EG030465	25.880	4.84
TcCBL3	Thecc1EG030580	25.986	4.83
TcCBL4	Thecc1EG015411	24.351	5.05
TcCBL5	Thecc1EG016487	25.129	4.60
TcCBL8	Thecc1EG016488	24.652	4.90
TcCBL10	Thecc1EG037279	29.393	4.87

Vitis venifera			
VvCBL1-1	GSVIVG01004840001	24.472	4.81
VvCBL1-2	GSVIVG01004842001	24.472	4.81
VvCBL3-1	GSVIVG01004678001	21.261	4.86
VvCBL3-2	GSVIVG01014744001	26.009	4.93
VvCBL4	GSVIVG01038549001	24.518	4.82
VvCBL5	GSVIVG01019554001	30.093	5.07
VvCBL8	GSVIVG01019555001	25.244	4.82
VvCBL10-1	GSVIVG01035369001	29.812	5.15
VvCBL10-2	GSVIVG01035370001	28.987	5.00
Zea mays			
ZmCBL3-1	GRMZM2G173424	25.911	4.98
ZmCBL3-2	GRMZM2G033680	25.707	5.01
ZmCBL3-3	GRMZM2G010093	25.661	4.96
ZmCBL4-1	GRMZM2G001221	23.967	4.87
ZmCBL4-2	GRMZM2G137751	24.582	4.86
ZmCBL4-3	GRMZM2G110080	24.582	4.86
ZmCBL9-1	GRMZM2G015324	24.453	4.79
ZmCBL9-2	GRMZM2G107575	24.471	4.89
ZmCBL10	GRMZM2G116584	35.849	5.46
