# Supplementary material for: Genome-wide identification of Calcineurin B-Like (CBL) gene family of plants reveals novel conserved motifs and evolutionary aspects in calcium signaling events
Source: BMC Plant Biol. 2015 Aug 6;15:189. doi: 10.1186/s12870-015-0543-0 (PMC4527274; doi:10.1186/s12870-015-0543-0)
Supplement: Additional file 4: — Table showing lists of OsCBL primers used during qRT-PCR analysis. Primers were designed using primer3 software (http://primer3.ut.ee/). [file 12870_2015_543_MOESM4_ESM.pdf]

Additional Data File 4

Table showing lists of OsCBL primers used during qRT-PCR analysis. Primers were designed using primer3 software (http://primer3.ut.ee/).

Gene Name	Locus ID	Forward primer	Reverse primer
OsCBL3-1	LOC_Os03g42840	CCCGTGCGCTCTCAGTATTTC	GTGTCGGCCTCCTCAAATGTC
OsCBL3-2	LOC_Os12g06510	ATTGACAAGGAGGAGTGGCG	ATGCGTCCTCAACCTGGGAG
OsCBL3-3	LOC_Os12g40510	CTGGTTCTTCGTCATCCCTC	CATCGACCTGGGAATGGAAG
OsCBL4-1	LOC_Os02g18880	TCGGGGAGTTTGTTCGATCC	CGATCTTCCCGTCGCTGTTT
OsCBL4-2	LOC_Os02g18930	GAGGCCCTCTTTGAGCTGTTC	GCTGAGGGATCGAACGAACTC
OsCBL4-3	LOC_Os05g45810	GAGCTCAGGGAGATGGTCTTGG	TGTTCCTTAGTGATGCCGGG
OsCBL9	LOC_Os10g41510	GGTTCTGTGATCGACGATGGG	CCTTGAGGTACGGAAGAGTC
OsCBL10-1	LOC_Os01g39770	GAGCGTGAGGAGGTTATGCA	TCAACCGCGGTGTTGAAGAC
OsCBL10-2	LOC_Os01g51420	TTGGAGTCAGAGGTGCAGCT	GAACGCCGCTGTAGTGTCCTTC
